# Looking through the Lens of the Ribosome Biogenesis Evolutionary History: Possible Implications for Archaeal Phylogeny and Eukaryogenesis

**DOI:** 10.1093/molbev/msac054

**Published:** 2022-03-11

**Authors:** Michael Jüttner, Sébastien Ferreira-Cerca

**Affiliations:** Regensburg Center for Biochemistry, Biochemistry III – Institute for Biochemistry, Genetics and Microbiology, University of Regensburg, Regensburg, Germany

**Keywords:** ribosome, ribosome biogenesis, archaea, Asgard, DPANN, Haloarchaea, Nanohaloarchaea, eukaryotes, halophily, unlinked rRNA genes, RNA, evolution, tree of life

## Abstract

Our understanding of microbial diversity and its evolutionary relationships has increased substantially over the last decade. Such an understanding has been greatly fueled by culture-independent metagenomics analyses. However, the outcome of some of these studies and their biological and evolutionary implications, such as the origin of the eukaryotic lineage from the recently discovered archaeal Asgard superphylum, is debated. The sequences of the ribosomal constituents are amongst the most used phylogenetic markers. However, the functional consequences underlying the analysed sequence diversity and their putative evolutionary implications are essentially not taken into consideration. Here, we propose to exploit additional functional hallmarks of ribosome biogenesis to help disentangle competing evolutionary hypotheses. Using selected examples, such as the multiple origins of halophily in archaea or the evolutionary relationship between the Asgard archaea and Eukaryotes, we illustrate and discuss how function-aware phylogenetic framework can contribute to refining our understanding of archaeal phylogeny and the origin of eukaryotic cells.

## Introduction: Ribosomes and the Discovery of Archaea

Ribosomes are complex RNA–protein assemblages responsible for translating genetic information encoded in messenger RNAs into proteins (Fox 2016). Ribosomes are universally conserved macromolecules and some of their structural components—the ribosomal RNA (rRNA) and ribosomal proteins (r-proteins)—are commonly used as phylogenetic markers (see below) (Melnikov et al. 2012; Petrov et al. 2014a, 2014b, 2015; Bowman et al. 2020). Although the ribosome, as a functional entity conducting the translation process, is universally present in any living cell, there are significant structural and compositional variations across and within the main taxonomic lineages: bacteria, archaea, and eukaryotes (Melnikov et al. 2012; Shasmal and Sengupta 2012; Hashem et al. 2013; Ban et al. 2014; Bowman et al. 2020; Penev et al. 2020; Tirumalai et al. 2020; Waltz et al. 2020; Stepanov and Fox 2021; Vicens et al. 2021). Likewise, ribosome biogenesis, the process by which ribosomal subunits are generated, shows substantial differences across, but also within, the main taxonomic groups (Thomson et al. 2013; Henras et al. 2015; Davis and Williamson 2017; Ferreira-Cerca 2017; Baßler and Hurt 2019; Klinge and Woolford 2019; Londei and Ferreira-Cerca 2021).

In the following, we will only introduce the general aspects of ribosome biology that are necessary for general comprehension of our viewpoint. For additional details on ribosome composition and ribosome biogenesis across the tree of life, several recent reviews are available to the curious reader (Thomson et al. 2013; Henras et al. 2015; Davis and Williamson 2017; Ferreira-Cerca 2017; Baßler and Hurt 2019; Klinge and Woolford 2019; Londei and Ferreira-Cerca 2021).

In brief, ribosomes can be divided into two ribosomal subunits, hereafter small subunit (SSU) and large subunit (LSU), respectively. Each ribosomal subunit is composed of rRNA and r-proteins, where the size and number of rRNA and r-proteins necessary for building the ribosomal subunits vary across the tree of life (Melnikov et al. 2012; Ban et al. 2014; Bowman et al. 2020). The ribosomes of most bacteria and archaea consist of the 16S rRNA (SSU) and 23S, 5S rRNAs (LSU), whereas the ribosomes of eukaryotes consist of the 18S rRNA (SSU) and LSU 25/28S, 5.8S, and 5S rRNAs (LSU). In addition to the universally conserved rRNA core, 33 r-proteins are universally conserved. Furthermore, a various number of domain-specific r-proteins, some of which are either only found in bacteria, archaea, or eukaryotes, or only shared between archaea and eukaryotes, are associated with the rRNA scaffolds (Melnikov et al. 2012; Armache et al. 2013; Ban et al. 2014; Ferreira-Cerca 2017; Londei and Ferreira-Cerca 2021). Accordingly, the prototype composition and structure of the ribosomal subunits differ between bacteria, archaea, and eukaryotes. However, from a structural and compositional point of view, the archaeal and eukaryotic mature ribosomal subunits are more similar than their bacterial counterparts, thereby indicating a closer association of the archaeal and eukaryotic ribosome evolutionary history (Melnikov et al. 2012; Armache et al. 2013; Ban et al. 2014; Ferreira-Cerca 2017; Londei and Ferreira-Cerca 2021).

Ribosome biogenesis represents one of the most energy-consuming processes within every cell, and it is tightly regulated and interconnected with other fundamental cellular processes, such as cell division or cellular growth (Gourse et al. 1996; Nomura 1999; Warner 1999; Bernstein et al. 2007; Freed et al. 2010; Teng et al. 2013; Bosdriesz et al. 2015; Prakash et al. 2019; Dai and Zhu 2020). It is, therefore, not very surprising that over the last two decades, several rare human disorders associated to ribosome synthesis malfunctions, collectively known as ribosomopathies, have been described (Freed et al. 2010; Teng et al. 2013; Danilova and Gazda 2015; Farley and Baserga 2016; Mills Eric and Rachel 2017; Calamita et al. 2018; Tahmasebi et al. 2018; Bohnsack and Bohnsack 2019; Da Costa et al. 2020; Kampen et al. 2020; Venturi and Montanaro 2020). Moreover, there is increasing evidence that ribosome biogenesis dysfunction may also promote cellular transformation and ageing (Sulima et al. 2017; Bustelo and Dosil 2018; Tahmasebi et al. 2018; Bohnsack and Bohnsack 2019; Penzo et al. 2019; Sulima et al. 2019; Turi et al. 2019). Accordingly, ribosome biogenesis occupies a global and prominent cellular role.

In addition to the structural components described previously, ribosomal subunits assembly also requires transiently acting factors, also known as ribosome biogenesis or assembly factors, facilitating the ribosomal subunit assembly process (Thomson et al. 2013; Henras et al. 2015; Davis and Williamson 2017; Ferreira-Cerca 2017; Baßler and Hurt 2019; Klinge and Woolford 2019; Londei and Ferreira-Cerca 2021). Strikingly, none of these factors, apart from the almost universally conserved dimethyl transferase ksgA/Dim1, are universally contributing to the ribosome biogenesis pathway (Seistrup et al. 2017; Knüppel et al. 2021). Although the number of ribosome biogenesis factors identified in bacteria (and presumably in archaea) is relatively modest (∼40–50), more than 200 ribosome biogenesis factors have been described in eukaryotes (Thomson et al. 2013; Henras et al. 2015; Davis and Williamson 2017; Ferreira-Cerca 2017; Baßler and Hurt 2019; Klinge and Woolford 2019; Londei and Ferreira-Cerca 2021). The lack of conservation at the level of ribosome biogenesis factors highlights the molecular diversity and plasticity of the ribosome biogenesis process across the tree of life. Still, it is important to note that a few ribosome biogenesis factors, most of which are critical for the cytoplasmic steps of eukaryotic ribosomal subunit maturation, are shared between archaea and eukaryotes (Ebersberger et al. 2014; Ferreira-Cerca 2017; Birikmen et al. 2021; Londei and Ferreira-Cerca 2021). Although the respective in vivo functions of these proteins are not yet well understood in archaea, recent studies suggest that some functional aspects of ribosome biogenesis might be shared between archaea and eukaryotes (Veith et al. 2012; Ferreira-Cerca 2017; Knüppel et al. 2018; Londei and Ferreira-Cerca 2021).

Hence, a better understanding of the evolutionary history of ribosome biogenesis and function can additionally contribute to portraying the evolutionary process across the tree of life and stimulate new avenues of research at the crossroads of evolution and molecular biology.

### Defining Archaea through the Ribosome Lens

Before going any further, it is important to briefly introduce the significance of the ribosome’s universal conservation and how exploiting knowledge about its conservation and diversity can provide insights into evolutionary relationships between organisms.

**fig. 1.**
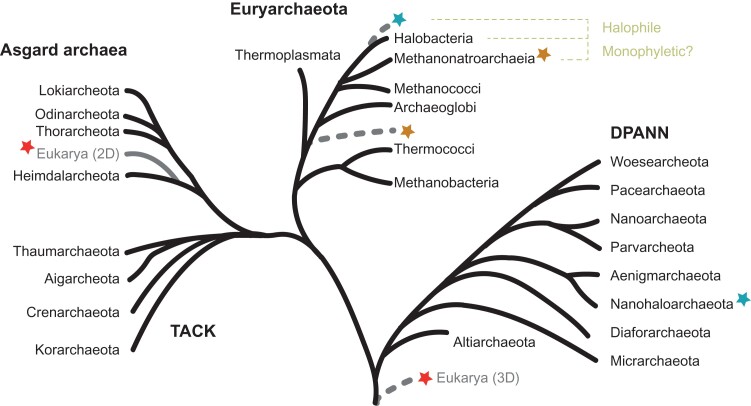
Archaeal diversity and their current phylogenetic relationship. A simplified archaeal phylogenetic tree based on Tahon et al. (2021) is depicted. Archaea are divided into the Euryarchaea phylum, and the TACK, DPANN, Asgard superphyla. Note that the exact position of the domain Eukarya (red star) as a sister group of or within the Asgard archaea is subject to discussion (see main text for details). Positioning of the Methanonatronarchaeia (orange star) within the Methanotecta superclass (Halobacteria–Archaeoglobi–Methanogens class II) or early branching before the emergence of the Archaeoglobi remains controversial (see main text for details). The relative positioning of the Nanohaloarchaea (blue star) within the DPANN superphylum or as a sister group of halobacteria and the DPANN phylogeny remains unstable (see main text for details). Branch lengths are not reflective of phylogenetic/evolutionary distance.

Ribosomes and the definition of archaea as an independent domain of life are entangled with each other, through the ground-breaking initial studies of Carl Woese and George Fox who meticulously generated and compared catalogs of rRNA fragments from diverse organisms (Fox et al. 1977; Woese and Fox 1977; Woese et al. 1990; Albers et al. 2013). The outcome of these analyses culminated by recognizing the archaea as an independent domain of life and classifying life on Earth into three domains of life (3D) comprising bacteria, archaea, and eukaryotes (Fox et al. 1977; Woese and Fox 1977; Woese et al. 1990; Albers et al. 2013) (see fig. 1). Although the rRNA molecules have played an initial role to support the independent archaeal phylogenetic placement, other biological processes, such as the analysis of archaeal multi-subunit DNA-dependent RNA polymerase by the Zillig group (Huet et al. 1983), or archaeal membrane biology spearheaded by Kandler and König (1978) should not be forgotten as instrumental discoveries additionally supporting the archaea as an independent domain (Kandler and König 1978; Huet et al. 1983; Woese et al. 1990; Albers et al. 2013).

However, it is also the same ribosomes that have challenged Carl Woese’s three-domain organization model. Particularly, the pioneering work of James Lake, who compared structural features of ribosomal subunits isolated from diverse organisms seen under the electron microscope, has challenged the emerging 3D organization view (Henderson et al. 1984; Lake et al. 1984; Lake 1985, 2015). The outcome of James Lake’s laboratory studies is best known under the “Eocyte hypothesis” which suggests that in contrast to the 3D model, eukaryotic cells were emerging from the archaeal domain (from the Crenarchaeota; see fig. 1). Thus, supporting a two domains view of the tree of life (Henderson et al. 1984; Lake et al. 1984; Lake 1985, 2015; Rivera and Lake 1992). Ironically, part of this hypothesis was drawn on initial observations that were made on phylogenetically diverse archaeal organisms, representing both Euryarchaeota (i.e., *Thermococcus celer* or *Thermoplasma acidophilum*) and Crenarchaeota, and which were collectively grouped as Eocytes (later on this definition was restricted to Crenarchaeota), whereas the remaining archaea where mostly containing Methanogens and Halobacteria (Lake et al. (1984) discussed in Gaia et al. (2018)). Despite these initial limitations and in agreement with Lake’s observations, it is now well established that the structures and compositions of archaeal ribosomal subunits are more closely related to their eukaryotic counterparts, and the similarities are even more pronounced between the Thaumarchaeota–Aigarchaeota–Crenarchaeota–Korarchaeota (TACK) superphylum and eukaryotes (Melnikov et al. 2012; Armache et al. 2013; Ban et al. 2014).

The “Eocyte hypothesis” has been challenged many times by improving the quality of phylogenetic analyses by using increasing numbers of universally conserved markers (such as r-proteins) and an ever-increasing number of characterized biological diversity, thereby improving taxa sampling and phylogenetic reconstructions (Gribaldo et al. 2010; Da Cunha et al. 2017, 2018; Gaia et al. 2018; Zhou et al. 2018; Cavalier-Smith and Chao 2020). However, the recent discovery of the Asgard phylum and their incorporation into phylogenetic analyses challenges anew the 3D organization and has reactivated the discussions on the tree of life topology and the origin of eukaryotes (Gribaldo et al. 2010; Williams et al. 2013; McInerney et al. 2014; Koonin 2015a, 2015b; Raymann et al. 2015; Spang et al. 2015, 2018; Da Cunha et al. 2017, 2018; Eme et al. 2017; Zaremba-Niedzwiedzka et al. 2017; Imachi et al. 2020; Liu et al. 2021) (see fig. 1).

The choice of the universally conserved markers is a notoriously difficult task, and these markers are to a large extent biased towards ribosomal subunit structural components or mature ribosome-associated factors. Consequently, to some extent, the tree of life reflects a rather incomplete, probably biased, evolutionary history seen through the lens of ribosome evolution. Moreover, due to methodological constraints of phylogenetic analysis, the ribosome evolutionary history is also a rather incomplete story, as it focuses only on the selected part of the (co)evolutionary history of some shared components, thereby neglecting a large part of the actual ribosome biological diversity. In addition, a phylogenetic analysis may not be sufficient to address the evolutionary history of the biological processes enabling the formation of these essential macromolecular machines, namely, the evolution of ribosome biogenesis. Finally, phylogenetic analyses often focus on sequence variation to generate evolutionary models that do not fully integrate the functional consequences and/or constraints underlying these variations, leading to an average aggregation of nonequivalent (co)evolutionary information.

There is no doubt that the current tools and evolutionary models are great instruments allowing unprecedented insights into phylogenetic relationships, cellular evolution, and biodiversity. However, complementary strategies and viewpoints may assist in bringing forward our general understanding of cellular evolution.

## Refining the Ribosome Evolutionary History Book: Can Hallmarks of Ribosome Biogenesis Provide Additional Insights into the Evolutionary History of Life?

In the following, using selected examples of timely relevant topics, such as the multiple origins of halophily in archaea, the general organization of the tree of life (see supplementary text 1 and 2, Supplementary Material online, respectively), and the link between the Asgard archaea and the eukaryotic lineage (below), we illustrate and discuss how the integration of additional structural and functional features related to ribosome biogenesis and function may help support or challenge different (competing) evolutionary hypotheses.

### On Asgard Archaea and the Origin of Eukaryotes

Recently, the discovery of organisms that define the Asgard phylum has renewed discussion on the origin of eukaryotes and the relative evolutionary positioning of archaea and eukaryotes (2D vs. 3D) (Koonin 2015a, 2015b; Spang et al. 2015; Da Cunha et al. 2017, 2018; Zaremba-Niedzwiedzka et al. 2017; Spang et al. 2018; Imachi et al. 2020; Liu et al. 2021) (see fig. 1). It is now beyond any doubt that the Asgard organisms encode specific genes shared with eukaryotes unseen in other archaea analysed, so far, and as such merit very particular attention.

From the very first draft genome to the discovery of many new Asgard members around the globe and the cultivation of the first Asgard representative, the presence of eukaryotic signature proteins, never seen in non-eukaryotic organisms before, and phylogenetic analyses have baptized the Asgard superphylum as the "missing link" between archaea and eukaryotes. This provided additional arguments in favor of James Lake’s original “Eocyte” hypothesis (Cox et al. 2008; Koonin 2015b, 2015b; Lake 2015; Raymann et al. 2015; Spang et al. 2015; Zaremba-Niedzwiedzka et al. 2017; Liu et al. 2021).

Expanding from the ribosome evolutionary history, we highlight possible characteristics of the ribosome biogenesis pathway and its organization that might help us in refining our view regarding the origin of eukaryotes and improve our knowledge on archaeal phylogeny.

One is the evolutionary origin of complex eukaryotic ribosome biogenesis which is mostly characterized by a larger amount of ribosome biogenesis factors (>200 established in eukaryotes vs. ∼40–50 in bacteria), most of which are not shared between eukaryotes and bacteria (Henras et al. 2015; Ferreira-Cerca 2017; Baßler and Hurt 2019; Klinge and Woolford 2019; Londei and Ferreira-Cerca 2021). In contrast, a portion of the eukaryotic ribosome biogenesis factors, around 40 sequence homologs out of the >200 known ribosome biogenesis factors in eukaryotes, are also present in most archaeal genomes (Ebersberger et al. 2014; Birikmen et al. 2021; Londei and Ferreira-Cerca 2021). These observations, in addition to recent functional studies, suggest that some specific aspects of ribosome biogenesis are shared between archaea and eukaryotes (Ferreira-Cerca 2017; Knüppel et al. 2018; Londei and Ferreira-Cerca 2021). Together, with the fact that archaea and eukaryotes uniquely share several (∼34) r-proteins (Ban et al. 2014; Londei and Ferreira-Cerca 2021), it suggests that the archaeal and eukaryotic ribosome biogenesis pathways have evolved on the basis of a common ancestral pathway (Ferreira-Cerca 2017; Londei and Ferreira-Cerca 2021). As such, defining the timing of (eukaryotic) ribosome biogenesis factors and r-proteins expansion may provide information on the shared evolutionary history of archaea and eukaryotes. In the light of our current knowledge, the ubiquitous distribution of most of these ribosome biogenesis factors in eukaryotes, and the absence of most of them in archaea, including the Asgard superphylum, suggest a major expansion in the last eukaryotic common ancestor (LECA; and probably not in the last archaeal and eukaryotic common ancestor; LAECA) (Ebersberger et al. 2014; Ferreira-Cerca 2017; Birikmen et al. 2021; Londei and Ferreira-Cerca 2021). The significant difference of ribosome biogenesis factors between archaea and eukaryotes still does not exclude a pre-expansion/diversification phase within the archaeal lineage, and the presence of the highest numbers of shared archaeal/eukaryotic ribosome biogenesis factors within specific archaeal groups may further support the positioning of certain archaeal phyla closer to eukaryotes. According to a recent analysis (Birikmen et al. 2021), the number of sequence homologs of ribosome biogenesis factors present in the Asgard phylum is slightly higher than that in most other archaea, particularly in *Ca.* Prometheoarchaeum syntrophicum; however, this difference remains moderate in its amplitude (55–67) (Birikmen et al. 2021). When considering the r-proteins, a total of 12 r-proteins are described to be specific to eukaryotes (Lecompte et al. 2002; Londei and Ferreira-Cerca 2021). Interestingly, two of them, namely eL22 and eL28, could be identified in some members of the Asgard phylum (Zaremba-Niedzwiedzka et al. 2017). As such, the additional presence of sequence homologs of eukaryotic ribosome biogenesis factors and/or r-proteins within the Asgard phylum further supports the close evolutionary relationship between the Asgard phylum and eukaryotic lineage.

In addition to the presence/absence analysis of orthologs, genome organization can provide relevant insights into dynamics that may reflect the evolutionary history of life forms from a genome structure perspective. Inspired by this general idea, we have focused on the peculiar rRNA genes organization as it might as well provide insights into evolutionary trajectories.

**fig. 2.**
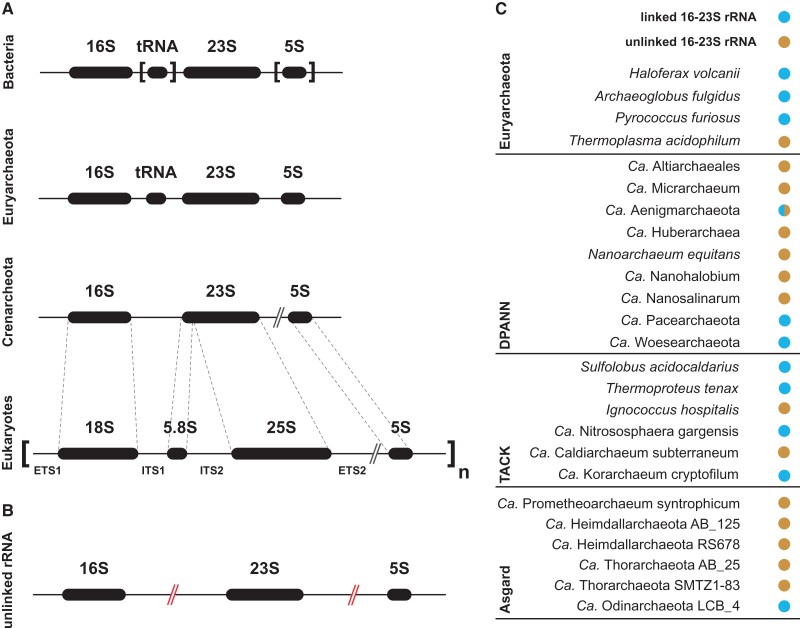
Unlinked rRNA genes in archaea. (*A*) Typical rRNA genes organization in bacteria, archaea, and eukaryotes. Typical rRNA genes transcriptional unit organization in bacteria, Euryarchaeota, Crenarchaeota, and eukaryotes are depicted. The evolutionary relationship of the different rRNA genes is depicted by dashed lines. The brackets indicate that the presence and/or position of the 5S and/or tRNA within the rRNA genes varies. The 5S rRNA can, in some cases, be unlinked from the main transcription unit as indicated by the //. ETS1: external transcribed spacer 1; ITS1: internal transcribed spacer 1; ETS2: external transcribed spacer 2; ITS2: internal transcribed spacer 2. (*B*) Unlinked rRNA genes organization. Unlinked archaeal rRNA genes organization, originally described in *T. acidophilum* (Tu and Zillig 1982), is schematically depicted and the rRNA gene separation is indicated by the red//. (*C*) Examples of linked and unlinked rRNA genes across archaea. Selected examples of linked and unlinked rRNA genes across archaea are depicted. Blue and brown dots indicate the presence of linked and unlinked 16S–23S rRNA genes, respectively. The presence of either linked or unlinked rRNA genes organization in different individual organisms classified as Aegnimarchaeota is indicated by a half blue/brown dot. Information on genome organization from the indicated organisms was obtained from NCBI, DOE-JGI, or eLSMG and are provided in detail in the accompanying supplementary information, Supplementary Material online. Note the strong prevalence of unlinked 16S–23S rRNA in the newly discovered Asgard and DPANN superphyla which further increase the so far recognized (Brewer et al. 2020) relative prevalence of unlinked rRNA genes in archaea. Note that the information provided does not reflect the overall quantitative prevalence of linked and unlinked rRNA genes across the archaea.

Ribosomal RNA genes are, in the vast majority of cellular contexts, forming a common transcriptional unit containing the SSU rRNA (16S/18S rRNA) and LSU RNA (23S/25S/28S—5.8S rRNAs), whereas bacteria and archaea usually possess 1–10 copies of these rRNA genes per haploid genome; in the vast majority of eukaryotes, these units are present in 100 to up to several 1,000 of tandemly repeated units distributed across one or several chromosomes (Hadjiolov 1985; Torres-Machorro et al. 2010; Symonová 2019). In this context, it is important to further highlight peculiarities of the evolution of the eukaryotic rRNA genes organization and, for example, the appearance of the additional 5.8S rRNA (Hadjiolov 1985). In fact, the eukaryotic specific 5.8S rRNA corresponds to the 5′ end of the bacterial/archaeal 23S rRNA which has been separated from the rest of the primitive eukaryotic LSU rRNA (Hadjiolov 1985), likely before tandem array expansion in the eukaryotic ancestor (see fig. 2*A*). However, please note that in microsporidian genomes the 5.8S rRNA is part of the rest of the 25S rRNA and the internal transcribed spacer 2 (ITS2) separating them is missing. This sequence organization and other peculiar rRNA features and the absence of mitochondria have been considered congruent with a very early origin of this eukaryotic lineage (Vossbrinck et al. 1987). However, the later discovery of the secondary loss of mitochondria in microsporidia suggests that these features are rather reflecting reductive evolution and adaption of the microsporidian translation machinery (Vossbrinck et al. 1987; Peyretaillade et al. 1998; Williams et al. 2002, 2021; Barandun et al. 2019; Nicholson et al. 2022). Another feature is the close physical presence of the 5S rRNA gene which despite being transcribed by a different polymerase in eukaryotes is an integral part of the repeated rDNA array in some eukaryotes (e.g., yeast) but has drifted apart during the evolution of eukaryotes, presumably after the 5.8S rRNA and formation of the ITS2, as it is scattered throughout genomes of most eukaryotes (Hadjiolov 1985; Torres-Machorro et al. 2010; Symonová 2019). Finally, expansion of the ITS2 spacer must have been accompanied by the recruitment/engineering of processing machinery enabling the proper processing of this spacer during rRNA maturation. In modern eukaryotes, several factors are required for this essential step of rRNA maturation (Baßler and Hurt 2019; Klinge and Woolford 2019; Zhang et al. 2020). To our knowledge, most of these factors are not found in known archaea (Ebersberger et al. 2014; Birikmen et al. 2021).

Archaea possess two main types of organizations of linked rRNA genes. The first type of cistronic unit contains the SSU and LSU rRNA separated by a tRNA (frequently tRNA^Ala^) in Euryarchaeota, an organization similar to the one observed in most bacteria. In the second type, no tRNA is separating the SSU and LSU rRNA. The 5S rRNA is, most of the time, part of the polycistronic unit and located downstream of the 23S rRNA but is an independent transcriptional unit in some organisms (Ferreira-Cerca 2017; Londei and Ferreira-Cerca 2021) (see fig. 2*A* and *B*). Finally, unlinked rRNA genes organization, whereby the SSU and LSU rRNA are transcriptionally separated from each other, has been observed in specific groups of bacteria (e.g., Deinococcus–Thermus phylum) and archaea (e.g., Thermoplasmatales order), and seems prevalent in symbiotic bacterial organisms (Tu and Zillig 1982; Borrel et al. 2014; Ahn et al. 2020; Brewer et al. 2020) (see fig. 2*B*). In eukaryotes, rRNA genes are predominantly linked and form cluster(s) of repeated sequences. These clusters can be found scattered on different chromosomes (Hadjiolov 1985; Torres-Machorro et al. 2010; Symonová 2019).

Overall, given the extent (in the number of archaeal organisms) of linked rRNA genes and the prevalence in the eukaryotic lineage of this organization, it appears more likely that LAECA contained linked rRNA genes. The presence of a tRNA between the 16S/23S rRNA in LAECA cannot be fully excluded; however, other parameters (such as shared r-proteins, e.g., rpS26/eS26) suggest a closer relationship of LAECA with the TACK superphylum, where the internal tRNA is typically absent (Brown et al. 1989; Yip et al. 2013; Brewer et al. 2020; Londei and Ferreira-Cerca 2021).

What about the Asgard phylum? Recently, using long-read sequencing technology, Brewer et al. (2020) noticed the presence of unlinked rRNA genes in the Asgardarcheota member analysed (Lokiarchaeum sp. GC14_75). Since in Eukaryotes, the rRNA genes are predominantly linked, we were intrigued by this observation. Linked rRNA genes are thought to provide an important means to coordinate the stoichiometric production of the two ribosomal subunits, which constitutes one of the largest energetic burdens of any growing cell (Liang and Fournier, 1997; Nomura, 1999, 2001; Warner, 1999).

Intrigued by the potential evolutionary consequences of this rRNA genes organization and to reveal how widespread is this organization across the Asgard phylum, we have (re)examined selected genomes across Archaea.

Analysis of the only cultivated Asgard archaeon, *Ca.* Prometheoarchaeum syntrophicum (Lokiarcheota) for which a complete genome is available (Imachi et al. 2020) also confirmed unlinked rRNA genes organization (see fig. 2*C* and supplementary information, Supplementary Material online). Intriguingly, the tRNA^Ala^ is located next to the 23S rRNA, in a similar fashion normally found in Euryarchaeota. To our surprise, unlinked rRNA genes are not restricted to Lokiarchaeota, as do found in Heimdallarchaeota, Wukongarchaeota, Njordarchaeota, and other recently described Asgard archaea we could examine (Zaremba-Niedzwiedzka et al. 2017; Liu et al. 2021; Xie et al. 2021; Wu et al. 2022) (see fig. 2*C* and supplementary information, Supplementary Material online). The only exception to what seems to be a shared feature among the Asgard archaea analysed is seen in Odinarchaeota (Odinarchaea LCB_4) (see supplementary information, Supplementary Material online). It should be noted that many of the Asgard phylum genomes available are genome assemblies obtained from metagenomics and that in these conditions, accurate rRNA genes assembly is known to be challenging (Yuan et al. 2015; Gruber-Vodicka et al. 2020). Accordingly, some of the evidence regarding unlinked/linked rRNA genes organization across archaea should be taken with some caution. However, it is interesting to note that both linked and unlinked rRNA genes organization in several Asgard archaea (e.g., Odin-/Loki-/Heimdallarchaeota) were identified from metagenome assembly (Zaremba-Niedzwiedzka et al. 2017). Moreover, the linked rRNA genes organization of Odinarchaea LCB_4 and unlinked rRNA genes organization in Heimdallarchaeota have been recently confirmed by long-read sequencing (Tamarit et al. 2021; Wu et al. 2022). Finally, the unlinked rRNA genes organization observed in Wukongarchaeota, Njordarchaeota (see supplementary information, Supplementary Material online) were obtained from independent metagenomic analyses (Liu et al. 2021; Xie et al. 2021).

Similar to Asgard archaea, many Diaforarchaeota–Parvarchaeota–Nanohaloarchaeota–Nanoarchaeota (DPANN) organisms, for which a few complete genomes and/or long-read sequencing are available, also do show unlinked rRNA genes organization as do a few members of the TACK superphylum (see fig. 2*C* and supplementary information, Supplementary Material online) (Brewer et al. 2020). As a side note, it has been previously observed that unlinked rRNA genes are more often found in symbiotic organisms (see Ahn et al. (2020); Brewer et al. (2020) and references therein). It is noteworthy that, *Ca.* Prometheoarchaeum syntrophicum could only be cultivated in coculture suggesting a certain degree of growth dependency (Imachi et al. 2020). Similarly, many DPANN organisms show unlinked rRNA genes (see fig. 2*C*) and only a few organisms have been amenable to cultivation, many of which only in coculture (Podar et al. 2008; Dombrowski et al. 2019; Hamm et al. 2019; Schwank et al. 2019; Sakai et al. 2022). Some well-established organisms with unlinked rRNA genes can be cultivated in pure culture, e.g., some members of the Thermoplasmatales (Darland et al. 1970). However, there is no evidence that these are nonobligate symbionts and that the cultivation conditions used might allow bypassing host requirements. It is not clear how unlinked rRNA genes and symbiosis are functionally interconnected. However, a recent study suggests that symbiosis may result in a less fluctuating environment and consequently a reduced need for complex transcriptional regulation. Hence in this condition, linked rRNA genes organization that normally contributes to stoichiometric production and energy optimization could be more easily rearranged into unlinked rRNA genes (Ahn et al. 2020).

Finally, the discovery of unlinked rRNA genes organization within the Asgard archaea has implications for the general organization of the Asgard phylum and our understanding of the origin of the eukaryotic lineage from Asgard archaea.

To our knowledge, most existing phylogeny analyses placing the eukaryotic lineage within the Asgard archaea, suggest a closer association of eukaryotes to Asgard possessing unlinked rRNA genes. They either occur as a sister group or within the Heimdallarchaeota–Gerdarchaeota–Kariarchaeota–Hodarchaeota–Wukongarchaeota–Njordarchaeota clades (Zaremba-Niedzwiedzka et al. 2017; Liu et al. 2021; Xie et al. 2021). A less well-supported phylogenetic hypothesis placed the eukaryotic lineage as a sister group to the Asgard and TACK superphyla (Liu et al. 2021). In the latter case, we assume that the last common ancestor of these lineages very likely possessed linked rRNA genes organization.

**fig. 3.**
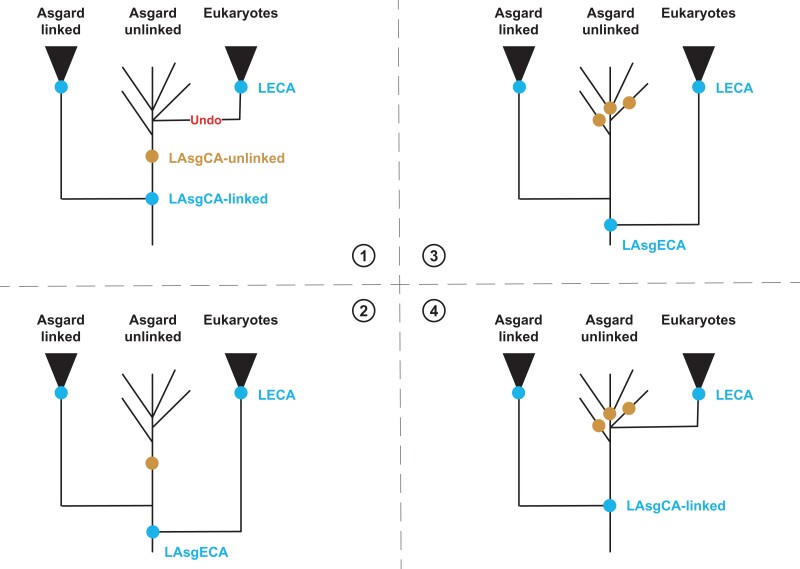
Evolutionary implications of rRNA genes organization for Asgard archaea and the origin of the eukaryotic lineage. Possible relationships between Asgard archaea and the eukaryotic lineages based on linked/unlinked rRNA genes repartition are indicated. The four depicted scenarios are discussed in detail in the main text. The presence of either linked (blue dot) or unlinked (brown dot) rRNA genes are indicated. Undo in scenario 1 indicates relinkage of unlinked rRNA genes. Note that linked rRNA genes are found in Odinarchaea LCB_4, whereas unlinked rRNA genes, seem to be prevalent in Asgard archaea and were observed in Lokiarchaeota, Thorarchaeota, Heimdallarchaeota, Wukongarchaeota, Njodarchaeota. LAsgECA, last Asgard-eukaryotic common ancestor; LECA, last eukaryotic common ancestor.

Taking into consideration the scenario where the eukaryotic lineage emerges from the Asgardarchaeota, we have tentatively illustrated the different evolutionary scenarios (fig. 3) and discuss in the following, the underlying biological consequences associated with rRNA genes organization.

The first possible scenario could be an emergence of the eukaryotic lineage from an Asgard ancestor containing unlinked rRNA genes, thereby implying early relinkage of the unlinked rRNA genes in the LECA (fig. 3—scenario 1). This “undo” scenario (rRNA genes relinkage) is in our opinion very unlikely in comparison to other possibilities. Intriguingly enough, to our knowledge, there is no known natural example of organisms with linked rRNA genes deriving from an ancestor with unlinked rRNA genes. “Unlinking” of rRNA genes does not seem to be challenging from a biological perspective and has been performed artificially in eukaryotes (Liang and Fournier 1997). In contrast, undoing this event (relinkage) might be difficult to functionally overcome after significant genome scrambling and putative partial elimination of key enzymes and/or rRNA elements involved in the maturation pathway (Grosjean et al. 2014; Ahn et al. 2020). The degeneration of the processing stem (see supplementary figure 4, Supplementary Material online) is an example in this context. Even though genetic systems might be unavailable or too immature in organisms with unlinked rRNA genes, performing genetic manipulation to relink rRNA genes in a cellular context where unlinked rRNA genes are naturally present could reveal the biological constraints of such an “undo” event for the formation of functional ribosomal subunits. Doing so might aid in efforts towards the phylogenetic placement of the eukaryotic lineage from unlinked rRNA genes Asgard ancestor.

The second main evolutionary scenario is the emergence of the eukaryotic lineage from a common Asgard ancestor with linked rRNA genes (fig. 3—scenarios 2–4). In this case, the placement of the eukaryotic lineage has important implications for the frequency of unlinked rRNA genes in the Asgard phylum (fig. 3). For instance, there are alternative models emerging where multiple independent unlinking or a unique unlinking event(s) may explain the observed distribution of rRNA genes organizations in this context (fig. 3). So far, the frequency of unlinking rRNA genes is unclear but the prevalence of this organization in closely related groups, such as the DPANN or Asgard phylum, is suggestive of common ancestry of this event at the phylum level. Based on this, the current phylogenetic relationship between Asgard and Eukaryotes or within the Asgard and/or DPANN phyla might need a revisit to better integrate this possibility. However, analyses of the evolution of genome organization will be necessary to reveal the relative timing of unlinking rRNA genes and offer better insights into its frequency.

The organization of the rRNA genes might provide limited but striking information on the tree of life topology, particularly the 2D/3D scenarios. The apparent prevalence of unlinked rRNA genes in Asgard archaea that might be associated with syntrophy/symbiosis and to some degree reductive evolution should remind us of the following. The modern Asgard archaea biology might have evolved diverging biological traits that are in part not related or significant to our understanding of eukaryogenesis. Moreover, the exact relationship between the Asgard superphylum and the eukaryotic lineage will require additional phylogenetic and compelling functional information to refine and understand the complex ancient evolutionary relationship of both lineages.

In summary, we believe that exploring the evolution of ribosome biogenesis, as an extension of the evolution of ribosomes often used in phylogenetic analyses, may provide means to refine phylogenetic relationships and evolutionary scenarios. This will further delineate the relevant biological traits and lifestyles of key cellular ancestors.

## Toward Functional Contextualization of Cellular Evolutionary History on Earth

Contextualization relates to considering something in a real environment, a process that can improve understanding beyond its isolation. For example, contextualization can facilitate language acquisition by putting new items/information into a known and meaningful situation, in opposition to only considering the word in its isolated form (Celce-Murcia and Olshtain 2000; Bax 2003). As languages evolve, words may fulfill a purpose at a defined time but also its meaning can vary depending on their context (Nowak and Krakauer 1999; Durkin 2009).

Similar to how the evolution of languages is studied, a systematic exploration of the situation-dependent biological meaning of sequence (and structure) variations may provide key information to our understanding of the cellular evolutionary history. In other words, sequence variations and their trajectories might be, on the one hand, reflecting natural sampling of functionally neutral or equivalent changes across the evolutionary landscape of fundamental processes, thereby providing insights into natural variability. Or they may, on the other hand, reflect inherited/acquired functional constraint(s) or adaptation(s) that to some extent, may have likely reshaped functional aspects of key biological processes, and restrict their diversity. To further use language analogy, selected words will not provide a meaningful sentence, rather a word cloud, that to some extent will be informative but may essentially ignore important semantic (biological) meaning.

Can functional contextualization be used to improve phylogenetic analysis? If yes, it is crucial to determine as many functional cornerstones as possible, understand and integrate their respective evolutionary history. Recently, we have tentatively coined this idea under the “functional phylogenetics” umbrella (Knüppel et al. 2021). In our view, functional phylogenetics aims to use experimentally validated functional information or information that may have key functional consequences to generate and test evolutionary scenarios, as exemplified above and is reminiscent of Forterre’s “biological plausibility arguments” (Forterre 2015). We believe that the evolutionary scenarios that can be deduced from phylogenetic analysis should take into consideration the functional meaning/outcome, when possible, of the nucleotides/amino-acids substitutions. In other words, whereas phylogenetic analyses score evolutionary distance based on sequence variations, functional phylogenetics would ideally score the underlying functional distance imposed by these changes. We believe that ribosome biogenesis and function are ideal to explore this idea. For now, this possibility is supported by a few manually curated features we have tentatively summarized herein. The number of examples needs to be ultimately increased, which is expected to improve our general knowledge on ribosome biogenesis and function in archaea substantially. We are also convinced that this general concept can be more broadly applied across various biological processes to extract relevant evolutionary information. Although complementary to phylogenetic analysis, the additional encoding of functional information (functional distance scoring) as biological constraints within an evolutionary framework, in a way similar to integrative structural biology (Ward et al. 2013), needs to be developed. Ultimately, functional phylogenetics would expand on the foundation of the phylogenetic approach. This would enable a more holistic approach towards understanding phylogenetic relationships that include information from variations in primary sequences and 2D, 3D structures. This approach may provide essential additional information to describe the relationship between all life forms on Earth and their respective evolutionary history.

We hope that the combined approaches and common curiosity will contribute to stimulate open discussions across fields and competence to solve what are probably some of the most exciting questions in biological science.

## Supplementary Material

msac054_Supplementary_Data
