# Supplementary material for: Looking through the Lens of the Ribosome Biogenesis Evolutionary History: Possible Implications for Archaeal Phylogeny and Eukaryogenesis
Source: Mol Biol Evol. 2022 Mar 11;39(4):msac054. doi: 10.1093/molbev/msac054 (PMC8997704; doi:10.1093/molbev/msac054)

Supplementary Information

Looking through the lens of the ribosome biogenesis evolutionary history: possible implications for archaeal phylogeny and eukaryogenesis

Michael Jüttner[1] and Sébastien Ferreira-Cerca[1*]

[1] Regensburg Center for Biochemistry, Biochemistry III – Institute for Biochemistry, Genetics and Microbiology, University of Regensburg, Universitätsstraße 31, 93053 Regensburg, Germany

[*] To whom correspondence should be addressed. Tel: +49 941 943 2539; Fax: +49 941 943 2474; Email: sebastien.ferreira-cerca@ur.de OrcId: https://orcid.org/0000-0002-0522-843X

This file contains:

- Supplementary Text 1 and 2
- Supplementary Figures 1-4
- Supplementary Note
- Supplementary Data Source: Selected example of rDNA genes organisation in archaea

Supplementary Text

Supplementary Text 1 | On the multiple origins of halophily in archaea

In the recent years, metagenomics-based genome assembly has revealed a plethora of hitherto unknown microorganisms that await their taxonomic classification (Spang et al. 2017; Castelle and Banfield 2018; Tahon et al. 2021 Sep 8). Among these findings, the discovery of nanosized archaea which have been grouped within the DPANN (Diapherotrites, Parvarchaeota, Aenigmarchaeota, Nanoarchaeota and Nanohaloarchaeota) superphylum is of particular interest (Williams et al. 2017; Dombrowski et al. 2019) (**Figure 1**). These organisms possess restricted metabolic potential and are believed to possess fast-evolving capacities, the latter feature making phylogenetic analysis rather difficult (Castelle et al. 2018; Dombrowski et al. 2019). Among these organisms, the Nanohaloarchaea are of major interest since their phylogenetic positioning is still unclear (**Figure 1**) (Narasingarao et al. 2012; Petitjean et al. 2014; Aouad et al. 2018; Hamm et al. 2019; Feng et al. 2021). On the one hand, Nanohaloarchaea have been clustered in the rather heterogenous DPANN group as a separate entity distinct from the "classical" haloarchaea (paraphyletic). On the other hand, some analyses position the Nanohaloarchaea and Haloarchaea (Halobacteria) with shared ancestry (monophyletic) (Narasingarao et al. 2012; Petitjean et al. 2014; Aouad et al. 2018) (**Figure 1**). The relative positioning of these two groups of organisms is critical from an evolutionary point of view, since in the first scenario halophily would have emerged (at least) twice independently (convergent evolution) and in the second one, halophily would be the product of a unique evolutionary event (Narasingarao et al. 2012; Petitjean et al. 2014; Aouad et al. 2018; Dombrowski et al. 2019). More recently, additional groups of halophilic organisms, the Methanonatronarchaeia and Hikarchaeia, have been described. Similarly, the current position of Methanonatronarchaeia also remains under active discussion (Sorokin et al. 2017; Aouad et al. 2018; Aouad et al. 2019; Sorokin et al. 2019; Martijn et al. 2020; Feng et al. 2021) (see **Figure 1**).

Interestingly, we have noticed common but also divergent traits related to ribosome biogenesis and function between Haloarchaea, Nanohaloarchaea, Methanonatronarchaeia and Hikarchaeia. Among these characteristics, helix 45 (h45), located at the 3´end of the 16S rRNA, contributes to the formation of the P-site of the SSU. Therefore, change in its structure/sequence is likely to alter ribosomal subunit function, and general cellular fitness (Formenoy et al. 1994; Knüppel et al. 2021). In addition, h45 is posttranscriptionally modified (Poldermans et al. 1979; van Gemen et al. 1987; Lafontaine et al. 1994: 1). These rRNA modifications contribute to the stabilisation of the SSU P-site (Van Charldorp et al. 1981; Demirci et al. 2010; Fischer et al. 2015). Recently we have reported that the dimethylation-dependent reaction of the almost universally conserved ksgA/Dim1 dimethyltransferase, on two universally conserved adenosines (A_{1518} and A_{1519}, or A_{1451} and A_{1452}, *E. coli* and *H. volcanii* numbering,

respectively) located in h45 is influenced by the overall sequence/architecture of h45 (Knüppel et al. 2021). Moreover, we could provide evidence that ksgA/Dim1 is required for archaeal cellular fitness, in good agreement with its particular relevance for ribosome biogenesis; ksgA/Dim1 is the only widespread conserved ribosome biogenesis factor (Ebersberger et al. 2014; Birikmen et al. 2021; Knüppel et al. 2021). Based on structural feature variation, we have classified h45 in archaea into 3 main types (**Supplementary Figure 1A**) (Knüppel et al. 2021). Type I represents the canonical and widespread structure. Type II differs from the canonical structure by the presence of a less stable G●U base pairing at the end of the h45 stem, thereby widening the modified GNRA tetraloop (see Supplementary **Figure 1A**) (Knüppel et al. 2021). In the case of type III, specific to Thermococcales, the internal G-U base pairing present in the h45 stem is replaced by a more stabilising G-C base pairing (**Supplementary Figure 1A** and below) (Formenoy et al. 1994; Knüppel et al. 2018). Interestingly, we could also observe that most organisms belonging to the Methanotecta superclass (Halobacteria, Archaeoglobi, Methanogens class II), and having a common ancestor, share similar h45 structure/sequence (classified as type II) and rRNA modification patterns, that differ from the prevalent modification pattern observed in h45-type I organisms (Knüppel et al. 2021) (Supplementary **Figure 1A-B**). Interestingly, the Nanohaloarchaea, and DPANN members, apart from the Altiarchaeota, do not show the h45 structure and/or sequence similarities with the group mentioned above (Knüppel et al. 2021). Similarly, the Methanonatronarchaeia do not contain the genuine type II-h45 architecture found in the Methanotecta group, but the type I architecture prevalent in most of the other Euryarchaeota and Crenarchaeota members, in agreement with their placement diverging before the Archaeoglobales (Sorokin et al. 2017; Aouad et al. 2018; Aouad et al. 2019; Sorokin et al. 2019) (see **Supplementary Figure 1A-B**). In contrast, the Hikarchaeota, are showing the classical Methanotecta h45 type II architecture, in agreement with their proposed phylogenetic placement (see **Figure 1**) (Martijn et al. 2020).

Our previous study also provides evidence that h45 sequence/structural variation, specifically observed in the Thermococcalles group (h45 type III) (Supplementary **Figure 1B**), is a poor substrate for the ksgA/Dim1 modification reactions in heterologous context, in comparison to the native Thermococcalles cellular context, thereby suggesting the presence of additional adaptation to compensate this single nucleotide sequence variation (Knüppel et al. 2021). Finally, previous mutation analyses of h45 also suggested the requirement of additional sequence/structural integrity of h45 stem for completion of the ksgA/Dim1-dependent modifications and optimal ribosomal function (Formenoy et al. 1994; Knüppel et al. 2021). This highlights the functional consequences and essential adaptations that might be necessary to tolerate sequence/structure changes within h45 observed during the course of evolution (see **Supplementary Figure 2**).

The structural/sequence diversity of h45 within the DPANN group is summarised in **Supplementary Figure 3**. Considering that h45 contributes to the formation of the P-site of the SSU and changes in its structure/sequences may significantly alter ribosomal subunit function, and general cellular fitness (Formenoy et al. 1994; Knüppel et al. 2021), the h45 evolution trajectories is likely not infinite and its potential diversity limited by its functional integrity and/or the capacity to evolve compensatory mechanisms. Intuitively the expectation of such functional constraints would likely result in limited diversity within closely related groups of organisms, as observed throughout the Methanotecta superclass. In other words, strong functional selection will more likely favour stepwise functionally neutral trajectories over deleterious ones (Nei 2005). Therefore, understanding the biological consequences of the possible evolutionary trajectories of key ribosomal subunit functional elements, like h45, may be an important resource to provide additional experimental evidence to the directionality of complex evolutionary history and support phylogenetic positioning.

In addition to h45, other rRNA elements are of interest to support putative evolutionary trajectories. For example, h31, another 16S rRNA elements contributing to the formation of the SSU P-site is worth investigating. Similar to h45, h31 is posttranscriptionally modified. Interestingly, the same relative residue (*E. coli* G_{966}, *H. volcanii* U_{910} and *S. cerevisiae* U_{1191}) is universally modified, however, the chemical nature of this rRNA modification differs between bacteria, and archaea/eukaryotes (Grosjean et al. 2008; Piekna-Przybylska et al. 2008; Ferreira-Cerca 2017). In *E. coli*, G_{966} is based-methylated by RsmD (Lesnyak et al. 2007). In contrast, in archaea and eukaryotes, specific addition of 3-amino-3-carboxypropyl (U-acp3) modification (U_{910} or G_{966}, *H. volcanii* and *E. coli* numbering, respectively) is performed by the archaeal and eukaryotic specific aminocarboxypropyltransferase Tsr3 (Twenty S rRNA accumulation 3) (Grosjean et al. 2008; Li et al. 2009; Meyer et al. 2016). During ribosome biogenesis, helix 31 is an important interaction hub that is sequentially associated with various ribosome biogenesis factors in eukaryotes, most of which have functional homologues in archaea (Wurm et al. 2010; Meyer et al. 2011; Meyer et al. 2016; Ferreira-Cerca 2017; Knüppel et al. 2018). Ultimately, the actions of these diverse factors facilitate the cooperative folding of different structures within the head domain of the SSU that are functionally crucial (Knüppel et al. 2018; Mitterer et al. 2019; Parker et al. 2019; Huang et al. 2020; Huang and Karbstein 2021; Plassart et al. 2021). Moreover, during translation elongation, h31 (among other helices) is described as a conserved structural pivot point driving ribosomal subunit motion induced by the specific binding of the universally conserved translation elongation factor EF-G (known as aEF2 and eEF2 in archaea and eukaryotes, respectively) (Paci and Fox 2015; Paci and Fox 2016).

Eukaryotic Tsr3 sequence homologues are widely distributed in archaea (Ebersberger et al. 2014; Birikmen et al. 2021) and the presence of h31 modification (U-acp3) has been established in *Haloferax*

volcanii (Grosjean et al. 2008). Interestingly, the DPANN group shows sequence/structure variation in this region, which might significantly alter the maturation process of this helix (**Supplementary Figure 1A and 1C**). Moreover, Tsr3 is not identified in most of these cellular contexts (Ebersberger et al. 2014; Mendler et al. 2019; Birikmen et al. 2021), thereby suggesting a similar evolutionary trajectory in the DPANN phylum of this functionally relevant region. In contrast, most of the Methanotecta superclass and the newly characterised Hikarchaeia show classical h31 structure/sequence and the presence of Tsr3 (see **Supplementary Figure 1A and 1C**) (Mendler et al. 2019). However, the Archaeoglobales and Methanonatroarchaeia seem to commonly lack Tsr3 (Mendler et al. 2019), and the Methanonatronarchaeia h31 additionally lacks the modified uridine at the expected position (see **Figure 1D**). Understanding functional consequences on Tsr3-dependent modification and ribosome function of this sequence/structural diversity will provide key information regarding the evolutionary plasticity of this region.

Another interesting example is the 330 Daltons rRNA modification of unknown chemical nature added by a yet unknown enzyme at the base of h44, on the third cytosine (C^{N330}) (C_{1404} or C_{1352}, *E. coli* and *H. volcanii* numbering, respectively) of three conserved consecutive cytosines (C_{1402-4} or $C_{1350-52}$, *E. coli* and *H. volcanii* numbering, respectively) (Grosjean et al. 2008). This rRNA modification has been at least observed in the bacterium *Thermotoga maritima*, and the archaeon *Haloferax volcanii*, but not in eukaryotes (Guymon et al. 2007; Grosjean et al. 2008). Whereas the modified cytosine (C_{1404} or C_{1352}, *E. coli* and *H. volcanii* numbering, respectively) is conserved in most archaea, it is mutated to a uridine in Nanohaloarchaea and some other DPANN members (see **Supplementary Figure 1A**). Whether the neighbouring cytosines (C_{1402-3} or $C_{1350-51}$, *E. coli* and *H. volcanii* numbering, respectively) can be modified instead or whether this modification is present at all, in this group of organisms, is not known. Although the role of the C^{N330} modification is not established, rRNA modifications and thus the respective enzymes are believed to fine-tune ribosome function (Decatur and Fournier 2002; Sloan et al. 2016 Dec 2). Therefore, perturbations of the substrate region might have significant consequences on cellular fitness and the evolutionary trajectories enabling this change may not be favourable. Again, in this context, the Nanohaloarchaea have a striking structural/sequence signature in a key strategical functional region of the SSU which more likely reflects a rather unique evolutionary history distinctive from other haloarchaea.

As illustrated above, the rRNA modifications and their respective molecular machinery, and very likely other fundamental aspects of ribosome biogenesis, are co-evolving with their respective rRNA substrates. Accordingly, the resulting evolutionary constraints and adaptations to maintain or modify these processes may help providing insights to support (or contradict) relative phylogenetic placement (see also discussion on the organisation of rRNA genes below). Particularly, the extent of shared

functional and structural converging features may provide additional arguments supporting the "functional plausibility" [see (Forterre 2015) and below] of unstable phylogenetic relationships, like e.g., the relative phylogenetic positioning of halophilic archaea discussed above. In our opinion, the examples mentioned are supportive of multiple origins of halophily.

Yet, going beyond the presented selected examples and expanding the functional evolutionary history towards more ribosomal constituents will be required to provide further insights into the archaeal evolutionary history. This endeavour is, however, mostly limited by our current understanding of ribosome biogenesis in archaea.

Supplementary Text 2 | On the monophyly of Archaea: 1D vs 2D vs 3D and the ribosome biogenesis viewpoint

Not only can sequence/structural variations within the rRNA coding region provide phylogenetic information but also the sequences surrounding the mature SSU and LSU rRNA, i.e., the external and internal transcribed spacers that are part of the rRNA genes transcriptional unit (see **Supplementary Figure 4**). These sequence elements participate in the ribosome biogenesis process and are therefore under functional selection (Liang and Fournier 1997; Thomson et al. 2013; Henras et al. 2015; Davis and Williamson 2017; Ferreira-Cerca 2017; Baßler and Hurt 2019; Klinge and Woolford 2019; Jüttner et al. 2020; Londei and Ferreira-Cerca 2021). The rRNA spacers sequences contain important structural and sequence information required for rRNA processing (Jüttner et al. 2020; Zhang et al. 2020). The rationale behind utilising rRNA spacer sequences is that these sequences tend to diversify faster than the rRNA itself, but the underlying variations are still under sufficient selective constraint that could provide phylogenetic information. For example, the sequence of eukaryotic ITS2 separating the 5.8S rRNA and the 25/28S rRNA is used in phylogenetic analysis of eukaryotes (Koetschan et al. 2010; Zhang et al. 2020). This idea has also been embraced by the archaeal field, at least in its early days, and has been explored by the group of Roger Garett (Kjems and Garrett 1990). In this early study, the emphasis was to establish structural models of rRNA spacer sequences at a time when the debate was more centred on establishing archaea as an independent domain of life. The secondary structure conservation analysis of the rRNA transcribed spacers has revealed that despite sequence diversity, these spacers adopt archaeal specific conserved structures that are distinguishable from the bacterial spacer structures (and the eukaryotic structures as well) (see **Supplementary Figure 4**). In other words, these analyses supported the monophyly of archaea (Kjems and Garrett 1990). Since the 1990s, a vast amount of completed genomes have become available and the time has probably come to revisit the sequence/structure information of these regions surrounding the rRNA and additionally employ RNA

structure-based phylogeny (Wolf et al. 2005; Lindgreen et al. 2006; Knies et al. 2008; Wolf et al. 2013). Furthermore, it is crucial to obtain information on the functional relevance and plasticity of these regions (Jüttner et al. 2020) and assess their utilisation in phylogenetic analysis.

The spacer sequences in modern eukaryotes have evolved in such a manner that any archaeal ancestry signal has been erased during the independent evolution of the eukaryotic lineage and the expansion of the eukaryotic ribosome biogenesis pathway. In the light of the newly defined Asgard archaea or DPANN, it is important to determine if these spacers regions are still structurally conserved across archaea, thereby further indicating a fundamental common trait of ribosome synthesis in archaea. We envision that the sequence/structure diversity of these flanking spacer regions may help stabilise phylogenetic relationships within archaea, like the origin of halophily and other still difficult phylogenetic placements (e.g., DPANN relationships).

In agreement with the work of the Garrett laboratory, the bacterial, archaeal and eukaryotic ribosomes and their ribosome biogenesis pathways are essentially distinguishable beyond any doubts as three different entities. They have more or less functional similarities that are reminiscent of 1) their respective close common ancestry, 2) similar inherited molecular constraints for which diverse molecular solutions have been (re)engineered at critical shared ancestral cellular nodes (Londei and Ferreira-Cerca 2021).

Recently, Cavalier-Smith (Cavalier-Smith and Chao 2020) and Devos (Devos 2021), have revisited a 1D model where archaea and eukarya would emerge from a common bacterial ancestor located within the PVC (Planctomycetes, Verrucomicrobia and Chlamydiae) superphylum, and strongly argue against previous criticisms that have challenged this concept (Cavalier-Smith and Chao 2020; Devos 2021). As mentioned above, only a sub-fraction of r-proteins are universally conserved and additional r-proteins are either specific to bacteria, archaea and/or eukaryotes, or only conserved between archaea and eukaryotes (Londei and Ferreira-Cerca 2021). Interestingly, although some r-proteins are non-homologous they are positioned into similar topological environments, thereby suggesting non-homologous convergent evolution (Forterre 2015; Cavalier-Smith and Chao 2020). From a ribosome biogenesis and function perspective, the main criticism of the 1D scenario is the non-homologous replacement of all the bacterial specific r-proteins (Forterre 2015; Cavalier-Smith and Chao 2020). In contrast, in a 2D/3D scenario, the universal ribosomal core (Melnikov et al. 2012) made by the universally conserved r-proteins would be essentially diversified into bacterial, archaeal and eukaryotic ribosomal subunits by acquisition/loss of r-proteins. In addition to previous arguments (Forterre 2015; Cavalier-Smith and Chao 2020), and unless assuming that all bacterial specific r-proteins were all functionally dispensable in the already evolved LUCA (Cavalier-Smith and Chao 2020), it is difficult to explain the absence of any remnants of these r-proteins in some cytosolic archaeal/eukaryotic

ribosomes. Similarly, it is equally difficult to explain the universal presence of functionally dispensable non-homologous r-proteins. Stepwise [see accretion model reviewed in (Petrov et al. 2015; Bowman et al. 2020)], convergent evolution stabilising important functional/structural features of the primordial ribosomal subunit, the universal core, appears in our opinion a more likely phenomenon of ribosome evolution.

Supplementary Figures

Supplementary Figure 1

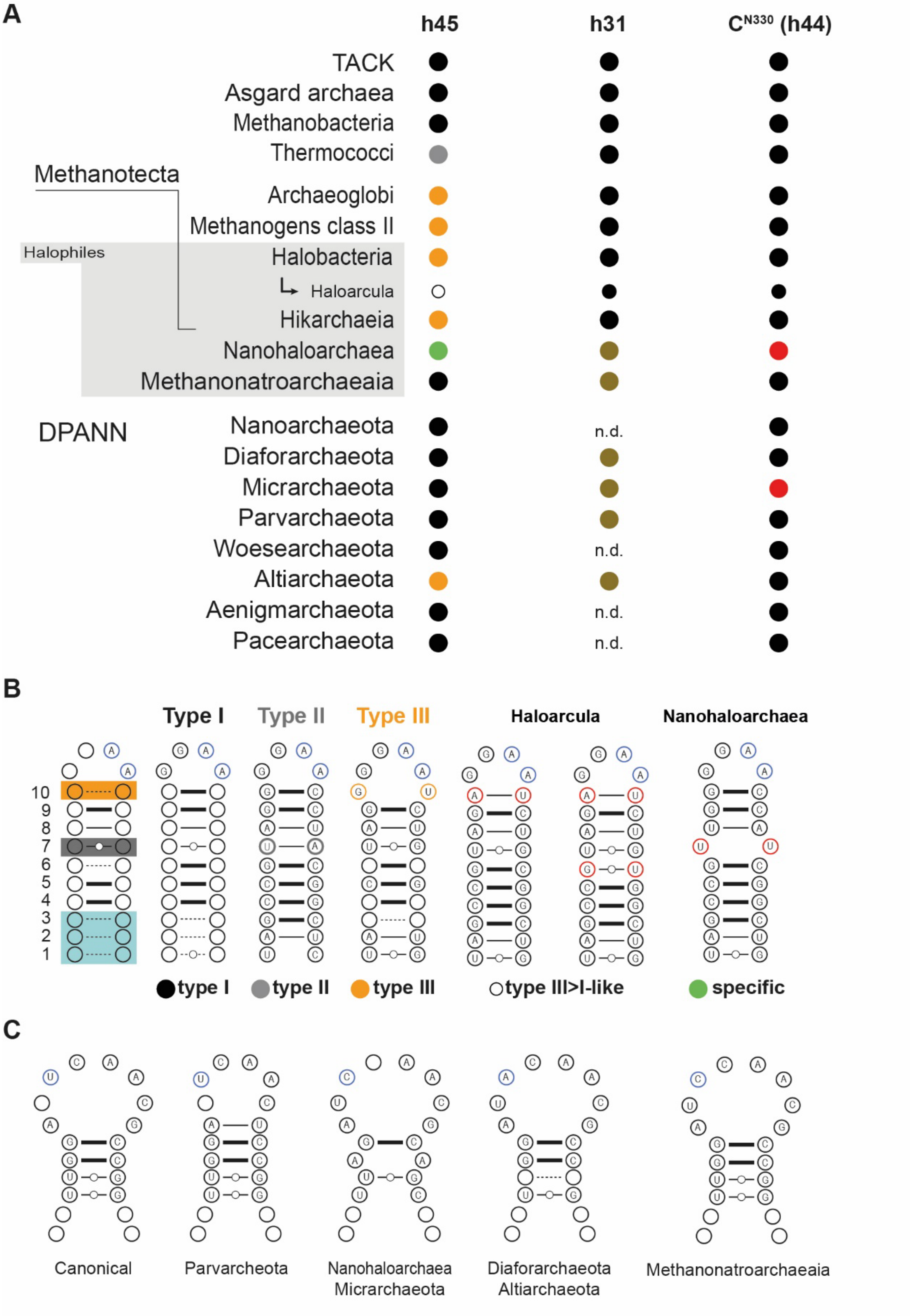

Supplementary Figure 1 | Examples of rRNA sequence/structure changes highlighting archaeal phylogenetic diversity.

A) Examples of structure/sequence diversity of selected SSU features across archaea.

Structure/sequence diversity of selected SSU structural features, like helix 45 and 31 (h45 and h31) or the C^{N330} modification at the base of helix 44 [C^{N330} (h44)] are depicted. Note that the chemical nature and enzyme responsible for the C^{N330} RNA modification of an apparent size of 330 Daltons located at a conserved cytosine (C^{1404} or C^{1352}, *E. coli* and *H. volcanii* numbering, respectively) at the base of h44 is currently unknown. Black dot indicates the "canonical" structure/sequence commonly found in archaea. For helix 45 grey, orange, green and open dots indicate h45 structures/sequence subtypes found in the respective group of organisms. For helix 31 and C^{N330} (h44) modification non-canonical features are marked in brown and red, respectively. The corresponding structure/sequence features for h45 and h31 are depicted in more detail in panels C and D, respectively.

n.d.: indicates that the structure/sequence of the corresponding feature could not be determined unambiguously. All representative 16S rRNA sequences were obtained from DOE-JGI, NCBI and/or the SILVA database.

B) **16S rRNA helix 45 secondary structure diversity across archaea.**

Archaea were classified into 3 major archaeal groups (type I, II and III) based on h45 sequence alignments and structure predictions of representative members of these groups (Knüppel et al. 2021). Base-pairing numbering (1-10) was annotated from the base of the h45 stem. When invariant the corresponding nucleotides and nature of the base-pairing (G-C larger line, A-U thinner line, or G●U dotted thinner line) are provided. Base pairing variability (G-C, A-U or G●U) is indicated by a dashed thinner line. An average representative archaeal h45 structure based on all the compiled sequences is depicted (upper left) (Knüppel et al. 2021). Note that the first 6 nucleotides forming the first 3 base pairs (indicated in blue) are relatively diverse in comparison to the almost invariant base-pairs at positions 4-6 and 8/9 across archaea. Variability at the G●U base-pairing at position 7 observed in type II and at position 10 specific to the type III group are indicated in grey and orange, respectively. Additional, exemplary structural sub-types restricted to a subset of archaeal organisms are provided. Figure panel is modified from (Knüppel et al. 2021). All representative 16S rRNA sequences were obtained from DOE-JGI, NCBI and/or the SILVA database and secondary structure predictions were performed using the Vienna RNA web suite (Gruber et al. 2008). See (Knüppel et al. 2021) for additional details.

C) 16S rRNA helix 31 secondary structure diversity across archaea.

Selected exemplary sequences and associated secondary structure predictions found in the indicated organisms are provided. Same as in panel C) secondary structure predictions were performed on selected 16S rRNA sequences as described above. Blue circled nucleotide indicates the position of the U-acp3 modification. Note the absence of uridine at this specific position in a subset of archaeal organisms.

Supplementary Figure 2

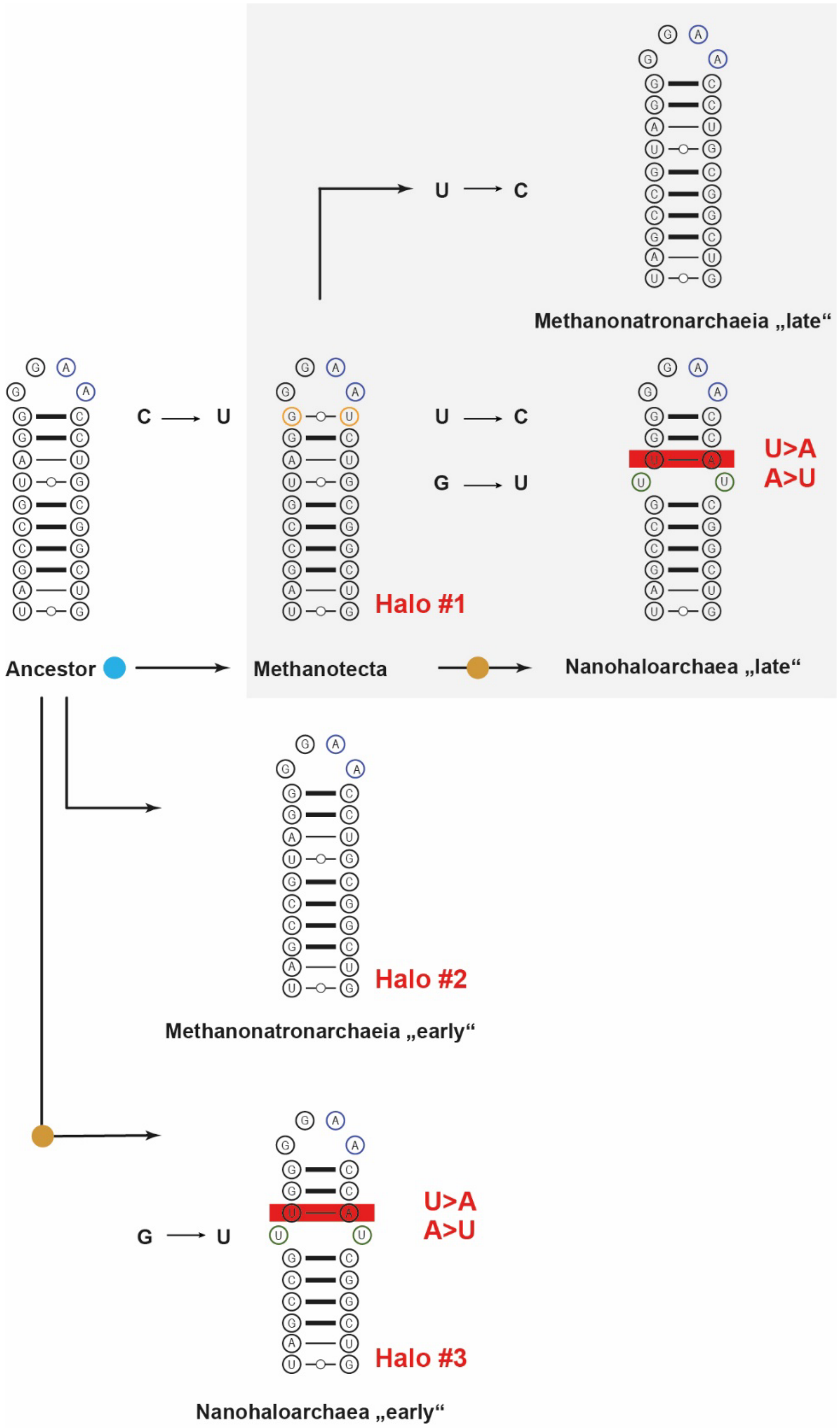

Supplementary Figure 2 |Putative evolutionary trajectories of h45 structure/sequence across halophilic archaea. Putative evolutionary trajectories of h45 from a common ancestor according to the different evolutionary scenarios of halophily origins. Note that the structure/sequence of the common ancestor is supported by the prevalence of this structure/sequence across archaea but may still be arbitrary. Necessary nucleotide exchanges are indicated. Note that in a monophyletic scenario of halophily, the Methanotecta hallmark (G-U base pairing at the tip of h45, indicated in orange) which modifies the ksgA/Dim1-dependent modification pattern (Knüppel et al. 2021), needs to be reversed multiple times to accommodate this evolutionary trajectory. The universally conserved and modified (demethylated) adenosines are indicated in blue. Base pairing flip A-U to U-A is underlined in red. The biological consequences of the h45 partial opening in Nanohaloarchaea are currently unknown. Blue and brown dots indicate the presence of linked and unlinked 16S-23S rRNA genes, respectively**.**

Supplementary Figure 3

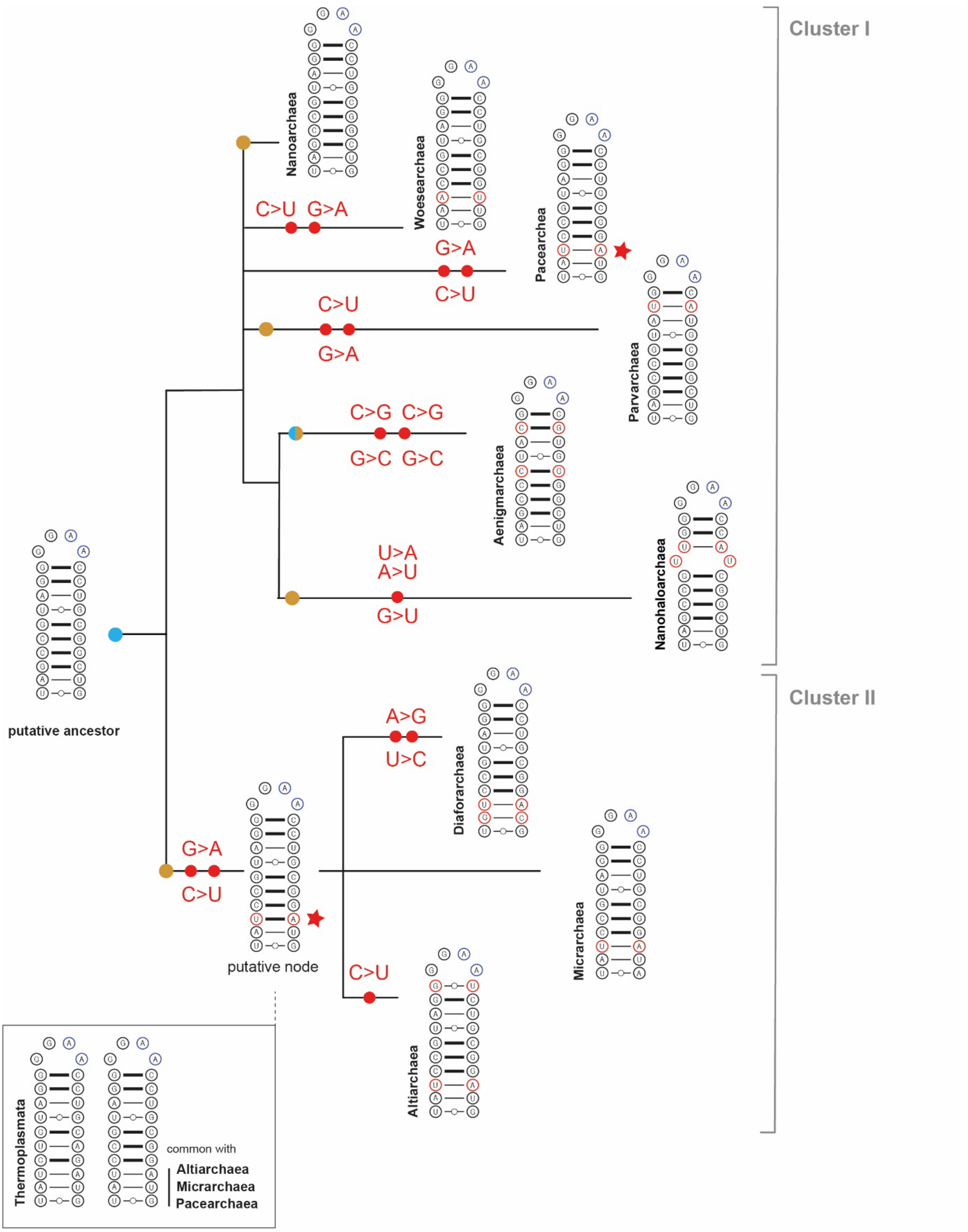

Supplementary Figure 3 | h45 structure/sequence variations across the DPANN superphylum. Putative evolutionary trajectories of h45 within the DPANN superphylum according to Dombrowski´s recent phylogeny (Dombrowski et al. 2020). Note that the structure/sequence of the common ancestor is supported by the prevalence of this structure/sequence across archaea but may be still arbitrary. Necessary nucleotide exchanges are indicated. Note the structure similarity of structure/sequence of the Pacearchaea and the common ancestor of Cluster II and Thermoplasmatales (red star), indicative of a common or convergent evolutionary history of the h45 structure/sequence. The universally conserved and modified adenosines are indicated in blue. Base pairing flip A-U to U-A is underlined in red. The biological consequences of the h45 partial opening in Nanohaloarchaea are currently unknown. Blue and brown dots indicate the presence of linked and unlinked 16S-23S rRNA genes, respectively**.** The presence of either linked or unlinked rRNA genes organisation in different individual organisms classified as Aegnimarchaea is indicated by a half blue/brown dot.

Supplementary Figure 4

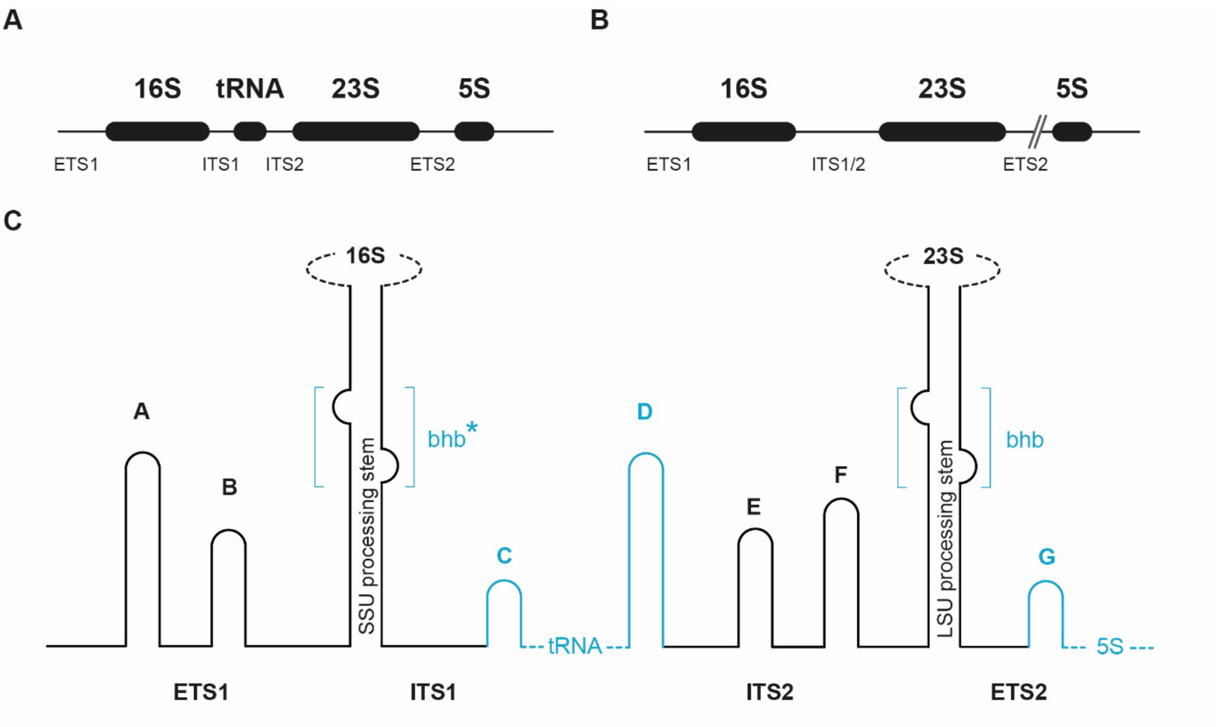

Supplementary Figure 4 | Typical rRNA genes organisation in archaea

A) Typical rRNA genes organisation in Euryarchaeota

Ribosomal RNA transcriptional unit architecture containing the 16S rRNA, tRNA, 23S rRNA and 5S rRNA separated by transcribed spacers typically found in Euryarchaeota is depicted.

B) Typical rRNA genes organisation in Crenarchaeota

Same as in panel A) except that the 16S and 23S rRNAs are not separated by a tRNA, and the 5S rRNA can, in some cases, be unlinked from the main transcriptional unit as indicated by the //.

C) Conserved structural elements found in the rRNA spacer sequences in archaea.

Schematic representation of conserved structural elements found in the rRNA spacers sequences, based on (Kjems and Garrett 1990), are depicted. Note the presence of helix A, B, E and F and the processing stems of the SSU and LSU in both Euryarchaeota and Crenarchaeota analysed, whereas helix C, D and G (indicated in blue) are presumably restricted to Euryarchaeota (Kjems and Garrett 1990). The processing stems are cleaved at the bulge-helix bulge motif (bhb) by the tRNA splicing endonuclease, a step required for the formation of archaeal-specific circular rRNA precursor intermediates (Jüttner et al. 2020; Qi et al. 2020 May 23; Schwarz et al. 2020). Note that the 16S bhb is absent or not well predicted in various archaea and is indicated with an * (Kjems and Garrett 1990; Jüttner et al. 2020; Qi et al. 2020 May 23). The size and the exact structure of the different helices vary between the organisms analysed (Kjems and Garrett 1990). ETS1: external transcribed spacer 1; ITS1: internal transcribed spacer 1; ETS2: external transcribed spacer 2; ITS2: internal transcribed spacer 2.

Supplementary References

Aouad M, Borrel G, Brochier-Armanet C, Gribaldo S. 2019. Evolutionary placement of Methanonatronarchaeia. Nature Microbiology. 4(4):558–559. doi:10.1038/s41564-019-0359-z.

Aouad M, Taib N, Oudart A, Lecocq M, Gouy M, Brochier-Armanet C. 2018. Extreme halophilic archaea derive from two distinct methanogen Class II lineages. Molecular Phylogenetics and Evolution. 127:46–54. doi:10.1016/j.ympev.2018.04.011.

Baßler J, Hurt E. 2019. Eukaryotic Ribosome Assembly. Annu Rev Biochem. 88(1):281–306. doi:10.1146/annurev-biochem-013118-110817.

Birikmen M, Bohnsack KE, Tran V, Somayaji S, Bohnsack MT, Ebersberger I. 2021. Tracing Eukaryotic Ribosome Biogenesis Factors Into the Archaeal Domain Sheds Light on the Evolution of Functional Complexity. Frontiers in Microbiology. 12:2598. doi:10.3389/fmicb.2021.739000.

Bowman JC, Petrov AS, Frenkel-Pinter M, Penev PI, Williams LD. 2020. Root of the Tree: The Significance, Evolution, and Origins of the Ribosome. Chem Rev. 120(11):4848–4878. doi:10.1021/acs.chemrev.9b00742.

Castelle CJ, Banfield JF. 2018. Major New Microbial Groups Expand Diversity and Alter our Understanding of the Tree of Life. Cell. 172(6):1181–1197. doi:10.1016/j.cell.2018.02.016.

Castelle CJ, Brown CT, Anantharaman K, Probst AJ, Huang RH, Banfield JF. 2018. Biosynthetic capacity, metabolic variety and unusual biology in the CPR and DPANN radiations. Nature Reviews Microbiology. 16(10):629–645. doi:10.1038/s41579-018-0076-2.

Cavalier-Smith T, Chao EE-Y. 2020. Multidomain ribosomal protein trees and the planctobacterial origin of neomura (eukaryotes, archaebacteria). Protoplasma. 257(3):621–753. doi:10.1007/s00709-019-01442-7.

Davis JH, Williamson JR. 2017. Structure and dynamics of bacterial ribosome biogenesis. Philos Trans R Soc Lond B Biol Sci. 372(1716):20160181. doi:10.1098/rstb.2016.0181.

Decatur WA, Fournier MJ. 2002. rRNA modifications and ribosome function. Trends in Biochemical Sciences. 27(7):344–351. doi:10.1016/S0968-0004(02)02109-6.

Demirci H, Murphy F, Belardinelli R, Kelley AC, Ramakrishnan V, Gregory ST, Dahlberg AE, Jogl G. 2010. Modification of 16S ribosomal RNA by the KsgA methyltransferase restructures the 30S subunit to optimize ribosome function. RNA. 16(12):2319–2324. doi:10.1261/rna.2357210.

Devos DP. 2021. Reconciling Asgardarchaeota Phylogenetic Proximity to Eukaryotes and Planctomycetes Cellular Features in the Evolution of Life. Molecular Biology and Evolution.(msab186). doi:10.1093/molbev/msab186. [accessed 2021 Feb 8]. https://doi.org/10.1093/molbev/msab186.

Dombrowski N, Lee J-H, Williams TA, Offre P, Spang A. 2019. Genomic diversity, lifestyles and evolutionary origins of DPANN archaea. FEMS Microbiology Letters. 366(2). doi:10.1093/femsle/fnz008. [accessed 2021 Jul 30]. https://doi.org/10.1093/femsle/fnz008.

Dombrowski N, Williams TA, Sun J, Woodcroft BJ, Lee J-H, Minh BQ, Rinke C, Spang A. 2020. Undinarchaeota illuminate DPANN phylogeny and the impact of gene transfer on archaeal evolution. Nature Communications. 11(1):3939. doi:10.1038/s41467-020-17408-w.

Ebersberger I, Simm S, Leisegang MS, Schmitzberger P, Mirus O, von Haeseler A, Bohnsack MT, Schleiff E. 2014. The evolution of the ribosome biogenesis pathway from a yeast perspective. Nucleic Acids Research. 42(3):1509–1523. doi:10.1093/nar/gkt1137.

Feng Y, Neri U, Gosselin S, Louyakis AS, Papke RT, Gophna U, Gogarten JP. 2021. The evolutionary origins of extreme halophilic Archaeal lineages. Genome Biology and Evolution.(evab166). doi:10.1093/gbe/evab166. [accessed 2021 Jul 30]. https://doi.org/10.1093/gbe/evab166.

Ferreira-Cerca S. 2017. Life and Death of Ribosomes in Archaea. In: Clouet-d'Orval B, editor. RNA Metabolism and Gene Expression in Archaea. Cham: Springer International Publishing. p. 129–158. https://doi.org/10.1007/978-3-319-65795-0_6.

Fischer N, Neumann P, Konevega AL, Bock LV, Ficner R, Rodnina MV, Stark H. 2015. Structure of the E. coli ribosome-EF-Tu complex at <3 A resolution by Cs-corrected cryo-EM. Nature. 520(7548):567–570.

Formenoy LJ, Cunningham PR, Nurse K, Pleij CWA, Ofengand J. 1994. Methylation of the conserved A1518-A1519 in Escherichia coli 16S ribosomal RNA by the ksgA methyltransferase is influenced by methylations around the similarly conserved U1512-G1523 base pair in the 3′ terminal hairpin. Biochimie. 76(12):1123–1128. doi:10.1016/0300-9084(94)90040-X.

Forterre P. 2015. The universal tree of life: an update. Frontiers in Microbiology. 6:717. doi:10.3389/fmicb.2015.00717.

van Gemen B, Koets H, Plooy C, Bodlaender J, Van Knippenberg P. 1987. Characterization of the ksgA gene of Escherichia coli determining kasugamycin sensitivity. Biochimie. 69(8):841–848. doi:10.1016/0300-9084(87)90210-0.

Grosjean H, Gaspin C, Marck C, Decatur WA, de Crécy-Lagard V. 2008. RNomics and Modomics in the halophilic archaea Haloferax volcanii: identification of RNA modification genes. BMC Genomics. 9:470–470. doi:10.1186/1471-2164-9-470.

Gruber AR, Lorenz R, Bernhart SH, Neuböck R, Hofacker IL. 2008. The Vienna RNA Websuite. Nucleic Acids Research. 36(suppl_2):W70–W74. doi:10.1093/nar/gkn188.

Guymon R, Pomerantz SC, Ison JN, Crain PF, McCloskey JA. 2007. Post-transcriptional modifications in the small subunit ribosomal RNA from Thermotoga maritima, including presence of a novel modified cytidine. RNA. 13(3):396–403. doi:10.1261/rna.361607.

Hamm JN, Erdmann S, Eloe-Fadrosh EA, Angeloni A, Zhong L, Brownlee C, Williams TJ, Barton K, Carswell S, Smith MA, et al. 2019. Unexpected host dependency of Antarctic Nanohaloarchaeota. Proc Natl Acad Sci USA. 116(29):14661. doi:10.1073/pnas.1905179116.

Henras AK, Plisson-Chastang C, O'Donohue M-F, Chakraborty A, Gleizes P-E. 2015. An overview of pre-ribosomal RNA processing in eukaryotes. WIREs RNA. 6(2):225–242. doi:10.1002/wrna.1269.

Huang H, Ghalei H, Karbstein K. 2020. Quality control of 40S ribosome head assembly ensures scanning competence. J Cell Biol. 219(11):e202004161. doi:10.1083/jcb.202004161.

Huang H, Karbstein K. 2021. Assembly factors chaperone ribosomal RNA folding by isolating helical junctions that are prone to misfolding. Proc Natl Acad Sci USA. 118(25):e2101164118. doi:10.1073/pnas.2101164118.

Jüttner M, Weiß M, Ostheimer N, Reglin C, Kern M, Knüppel R, Ferreira-Cerca S. 2020. A versatile cis-acting element reporter system to study the function, maturation and stability of ribosomal RNA mutants in archaea. Nucleic Acids Res. 48(4):2073–2090. doi:10.1093/nar/gkz1156.

Kjems J, Garrett RA. 1990. Secondary structural elements exclusive to the sequences flanking ribosomal RNAs lend support to the monophyletic nature of the archaebacteria. Journal of Molecular Evolution. 31(1):25–32. doi:10.1007/BF02101789.

Klinge S, Woolford JL. 2019. Ribosome assembly coming into focus. Nature Reviews Molecular Cell Biology. 20(2):116–131. doi:10.1038/s41580-018-0078-y.

Knies JL, Dang KK, Vision TJ, Hoffman NG, Swanstrom R, Burch CL. 2008. Compensatory Evolution in RNA Secondary Structures Increases Substitution Rate Variation among Sites. Molecular Biology and Evolution. 25(8):1778–1787. doi:10.1093/molbev/msn130.

Knüppel R, Christensen RH, Gray FC, Esser D, Strauß D, Medenbach J, Siebers B, MacNeill SA, LaRonde N, Ferreira-Cerca S. 2018. Insights into the evolutionary conserved regulation of Rio ATPase activity. Nucleic Acids Research. 46(3):1441–1456. doi:10.1093/nar/gkx1236.

Knüppel R, Trahan C, Kern M, Wagner A, Grünberger F, Hausner W, Quax TEF, Albers S-V, Oeffinger M, Ferreira-Cerca S. 2021. Insights into synthesis and function of KsgA/Dim1-dependent rRNA modifications in archaea. Nucleic Acids Res. 49(3):1662–1687. doi:10.1093/nar/gkaa1268.

Koetschan C, Förster F, Keller A, Schleicher T, Ruderisch B, Schwarz R, Müller T, Wolf M, Schultz J. 2010. The ITS2 Database III—sequences and structures for phylogeny. Nucleic Acids Research. 38(suppl_1):D275–D279. doi:10.1093/nar/gkp966.

Lafontaine D, Delcour J, Glasser A-L, Desgres J, Vandenhaute J. 1994. The DIM1 Gene Responsible for the Conserved m62Am62A Dimethylation in the 3′-Terminal Loop of 18 S rRNA is Essential in Yeast. Journal of Molecular Biology. 241(3):492–497. doi:10.1006/jmbi.1994.1525.

Lesnyak DV, Osipiuk J, Skarina T, Sergiev PV, Bogdanov AA, Edwards A, Savchenko A, Joachimiak A, Dontsova OA. 2007. Methyltransferase That Modifies Guanine 966 of the 16 S rRNA: FUNCTIONAL IDENTIFICATION AND TERTIARY STRUCTURE. The Journal of biological chemistry. 282(8):5880–5887. doi:10.1074/jbc.M608214200.

Li Z, Lee I, Moradi E, Hung N-J, Johnson AW, Marcotte EM. 2009. Rational extension of the ribosome biogenesis pathway using network-guided genetics. PLoS Biol. 7(10):e1000213–e1000213. doi:10.1371/journal.pbio.1000213.

Liang W-Q, Fournier MJ. 1997. Synthesis of functional eukaryotic ribosomal RNAs in trans: Development of a novel in vivo rDNA system for dissecting ribosome biogenesis. Proceedings of the National Academy of Sciences of the United States of America. 94(7):2864–2868.

Lindgreen S, Gardner PP, Krogh A. 2006. Measuring covariation in RNA alignments: physical realism improves information measures. Bioinformatics. 22(24):2988–2995. doi:10.1093/bioinformatics/btl514.

Londei P, Ferreira-Cerca S. 2021. Ribosome Biogenesis in Archaea. Frontiers in Microbiology. 12:1476. doi:10.3389/fmicb.2021.686977.

Martijn J, Schön ME, Lind AE, Vosseberg J, Williams TA, Spang A, Ettema TJG. 2020. Hikarchaeia demonstrate an intermediate stage in the methanogen-to-halophile transition. Nat Commun. 11(1):5490–5490. doi:10.1038/s41467-020-19200-2.

Melnikov S, Ben-Shem A, Garreau de Loubresse N, Jenner L, Yusupova G, Yusupov M. 2012. One core, two shells: bacterial and eukaryotic ribosomes. Nat Struct Mol Biol. 19(6):560–567. doi:10.1038/nsmb.2313.

Mendler K, Chen H, Parks DH, Lobb B, Hug LA, Doxey AC. 2019. AnnoTree: visualization and exploration of a functionally annotated microbial tree of life. Nucleic Acids Research. 47(9):4442–4448. doi:10.1093/nar/gkz246.

Meyer B, Wurm JP, Kötter P, Leisegang MS, Schilling V, Buchhaupt M, Held M, Bahr U, Karas M, Heckel A, et al. 2011. The Bowen–Conradi syndrome protein Nep1 (Emg1) has a dual role in eukaryotic ribosome biogenesis, as an essential assembly factor and in the methylation of Ψ1191 in yeast 18S rRNA. Nucleic Acids Research. 39(4):1526–1537. doi:10.1093/nar/gkq931.

Meyer B, Wurm JP, Sharma S, Immer C, Pogoryelov D, Kötter P, Lafontaine DLJ, Wöhnert J, Entian K-D. 2016. Ribosome biogenesis factor Tsr3 is the aminocarboxypropyl transferase responsible for 18S rRNA hypermodification in yeast and humans. Nucleic Acids Research. 44(9):4304–4316. doi:10.1093/nar/gkw244.

Mitterer V, Shayan R, Ferreira-Cerca S, Murat G, Enne T, Rinaldi D, Weigl S, Omanic H, Gleizes P-E, Kressler D, et al. 2019. Conformational proofreading of distant 40S ribosomal subunit maturation events by a long-range communication mechanism. Nat Commun. 10(1):2754–2754. doi:10.1038/s41467-019-10678-z.

Narasingarao P, Podell S, Ugalde JA, Brochier-Armanet C, Emerson JB, Brocks JJ, Heidelberg KB, Banfield JF, Allen EE. 2012. De novo metagenomic assembly reveals abundant novel major lineage of Archaea in hypersaline microbial communities. The ISME Journal. 6(1):81–93. doi:10.1038/ismej.2011.78.

Nei M. 2005. Selectionism and Neutralism in Molecular Evolution. Molecular Biology and Evolution. 22(12):2318–2342. doi:10.1093/molbev/msi242.

Paci M, Fox GE. 2015. Major centers of motion in the large ribosomal RNAs. Nucleic Acids Research. 43(9):4640–4649. doi:10.1093/nar/gkv289.

Paci M, Fox GE. 2016. Centers of motion associated with EF-Tu binding to the ribosome. RNA Biol. 13(5):524–530. doi:10.1080/15476286.2015.1114204.

Parker MD, Collins JC, Korona B, Ghalei H, Karbstein K. 2019. A kinase-dependent checkpoint prevents escape of immature ribosomes into the translating pool. PLoS Biol. 17(12):e3000329–e3000329. doi:10.1371/journal.pbio.3000329.

Petitjean C, Deschamps P, López-García P, Moreira D. 2014. Rooting the domain archaea by phylogenomic analysis supports the foundation of the new kingdom Proteoarchaeota. Genome Biol Evol. 7(1):191–204. doi:10.1093/gbe/evu274.

Petrov AS, Gulen B, Norris AM, Kovacs NA, Bernier CR, Lanier KA, Fox GE, Harvey SC, Wartell RM, Hud NV, et al. 2015. History of the ribosome and the origin of translation. Proc Natl Acad Sci USA. 112(50):15396. doi:10.1073/pnas.1509761112.

Piekna-Przybylska D, Decatur WA, Fournier MJ. 2008. The 3D rRNA modification maps database: with interactive tools for ribosome analysis. Nucleic Acids Research. 36(suppl 1):D178–D183. doi:10.1093/nar/gkm855.

Plassart L, Shayan R, Montellese C, Rinaldi D, Larburu N, Pichereaux C, Froment C, Lebaron S, O'Donohue M-F, Kutay U, et al. 2021. The final step of 40S ribosomal subunit maturation is controlled by a dual key lock. Elife. 10:e61254. doi:10.7554/eLife.61254.

Poldermans B, Roza L, Van Knippenberg PH. 1979. Studies on the function of two adjacent N6,N6-dimethyladenosines near the 3' end of 16 S ribosomal RNA of Escherichia coli. III. Purification and properties of the methylating enzyme and methylase-30 S interactions. Journal of Biological Chemistry. 254(18):9094–9100.

Qi L, Li J, Jia J, Yue L, Dong X. 2020 May 23. Comprehensive analysis of the pre-ribosomal RNA maturation pathway in a methanoarchaeon exposes the conserved circularization and linearization mode in archaea. RNA Biology.:1–15. doi:10.1080/15476286.2020.1771946.

Schwarz TS, Berkemer SJ, Bernhart SH, Weiß M, Ferreira-Cerca S, Stadler PF, Marchfelder A. 2020. Splicing Endonuclease Is an Important Player in rRNA and tRNA Maturation in Archaea. Front Microbiol. 11:594838–594838. doi:10.3389/fmicb.2020.594838.

Sloan KE, Warda AS, Sharma S, Entian K-D, Lafontaine DLJ, Bohnsack MT. 2016 Dec 2. Tuning the ribosome: The influence of rRNA modification on eukaryotic ribosome biogenesis and function. RNA Biology.:1–16. doi:10.1080/15476286.2016.1259781.

Sorokin DY, Makarova KS, Abbas B, Ferrer M, Golyshin PN, Galinski EA, Ciorda S, Mena MC, Merkel AY, Wolf YI, et al. 2019. Reply to 'Evolutionary placement of Methanonatronarchaeia.' Nature Microbiology. 4(4):560–561. doi:10.1038/s41564-019-0358-0.

Sorokin DY, Makarova KS, Abbas B, Ferrer M, Golyshin PN, Galinski EA, Ciordia S, Mena MC, Merkel AY, Wolf YI, et al. 2017. Discovery of extremely halophilic, methyl-reducing euryarchaea provides insights into the evolutionary origin of methanogenesis. Nat Microbiol. 2:17081–17081. doi:10.1038/nmicrobiol.2017.81.

Spang A, Caceres EF, Ettema TJG. 2017. Genomic exploration of the diversity, ecology, and evolution of the archaeal domain of life. Science. 357(6351):eaaf3883. doi:10.1126/science.aaf3883.

Tahon G, Patricia Geesink, Ettema TJG. 2021 Sep 8. Expanding Archaeal Diversity and Phylogeny: Past, Present, and Future. Annu Rev Microbiol. doi:10.1146/annurev-micro-040921-050212. [accessed 2021 Aug 6]. https://doi.org/10.1146/annurev-micro-040921-050212.

Thomson E, Ferreira-Cerca S, Hurt E. 2013. Eukaryotic ribosome biogenesis at a glance. J Cell Sci. 126(21):4815. doi:10.1242/jcs.111948.

Van Charldorp R, Heus HA, Knippenberg PHV, Joordens J, De Bruin SH, Hilbers CW. 1981. Destabilization of secondary structure in 16S ribosomal RNA by dimethylation of two adjacent adenosines. Nucleic Acids Research. 9(17):4413–4422. doi:10.1093/nar/9.17.4413.

Williams TA, Szöllősi GJ, Spang A, Foster PG, Heaps SE, Boussau B, Ettema TJG, Embley TM. 2017. Integrative modeling of gene and genome evolution roots the archaeal tree of life. Proc Natl Acad Sci USA. 114(23):E4602. doi:10.1073/pnas.1618463114.

Wolf M, Chen S, Song J, Ankenbrand M, Müller T. 2013. Compensatory Base Changes in ITS2 Secondary Structures Correlate with the Biological Species Concept Despite Intragenomic Variability in ITS2 Sequences – A Proof of Concept. PLOS ONE. 8(6):e66726. doi:10.1371/journal.pone.0066726.

Wolf M, Friedrich J, Dandekar T, Müller T. 2005. CBCAnalyzer: Inferring Phylogenies Based on Compensatory Base Changes in RNA Secondary Structures. In Silico Biology. 5(3):291–294.

Wurm JP, Meyer B, Bahr U, Held M, Frolow O, Kötter P, Engels JW, Heckel A, Karas M, Entian K-D, et al. 2010. The ribosome assembly factor Nep1 responsible for Bowen–Conradi syndrome is a pseudouridine-N1-specific methyltransferase. Nucleic Acids Research. 38(7):2387–2398. doi:10.1093/nar/gkp1189.

Zhang W, Tian W, Gao Z, Wang G, Zhao H. 2020. Phylogenetic Utility of rRNA ITS2 Sequence-Structure under Functional Constraint. International Journal of Molecular Sciences. 21(17). doi:10.3390/ijms21176395.

Supplementary Note

Relative distribution of linked and unlinked rRNA genes organisation in archaea

It is currently difficult to accurately assess the numbers and distribution of unlinked and linked rRNA genes organisation across archaea. Some of the current limitations to do so are highlighted below:

1) The quality of the data analysed

As mentioned in the main text rRNA genes assembly are particularly challenging when performed from short-read metagenomics datasets. High quality complete (closed) genomes ideally obtained from enriched/pure cultures or long reads sequencing technologies provide more accurate genomes assembly. However, the amounts of such genomes remain limited for archaea.

2) Representativity of the available data

The available data may be biased due to scientific focus and feasibility. These parameters influence sampling strategies, like the type of sampled environments and ultimately the type of data that are available (or missing). High-quality genomes assemblies are also predominantly available for organisms that are easily amenable to enrichment/pure cultures.

The relative distribution across archaea of linked/unlinked rRNA genes organisation can be assessed based on the recent study of Brewer and collaborators.

This study has analysed rRNA genes organisation using two types of data: those available at NCBI (up to January 2019) and long-reads sequencing from different types of selected environments.

According to this study, less than 5 % of the TACK superphylum (~75 total analysed) and slightly more than 5% in Euryarchaeota (~200 total analysed) of the “complete” genomes deposited in the NCBI database are unlinked (>12,000 genomes of bacteria and archaea were analysed, archaea representing a very small portion of these).

In the same study and based on long-read sequencing data: 50% of the TACK (46 total analysed) and slightly more than 5% of the Euryarchaeota (433 in total analysed) have unlinked rRNA genes organisation.

If combining both datasets, around 22% of the TACK superphylum and 7.5% of the Euryarchaeota analysed have unlinked rRNA genes.

From this example, it is expected that these relative numbers will be substantially revised in the coming years. However, large amounts of high-quality data across diverse biotopes will be necessary to minimize possible distribution bias.

Supplementary Data Source: examples of unlinked and linked rRNA genes organisation in archaea.

Candidatus Prometheoarchaeum syntrophicum strain MK-D1 chromosome, complete genome

Source GenBank: CP042905.1

16S rRNA

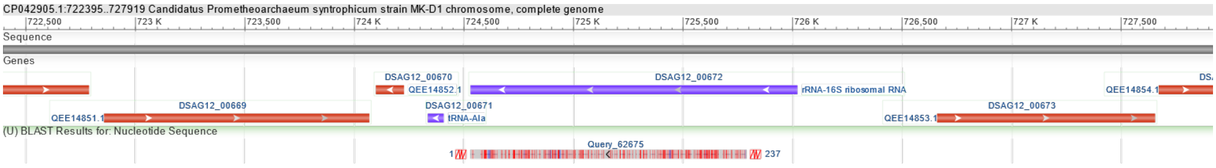

23S rRNA

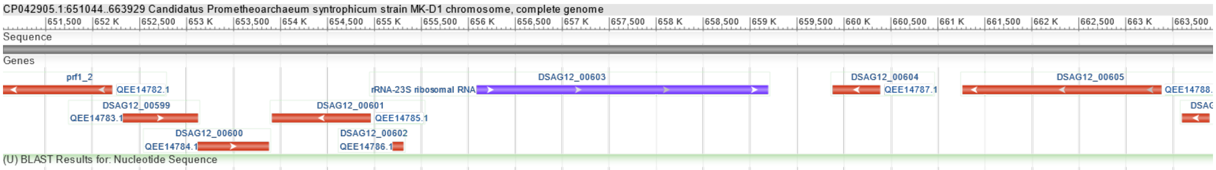

16S and 23S rRNA

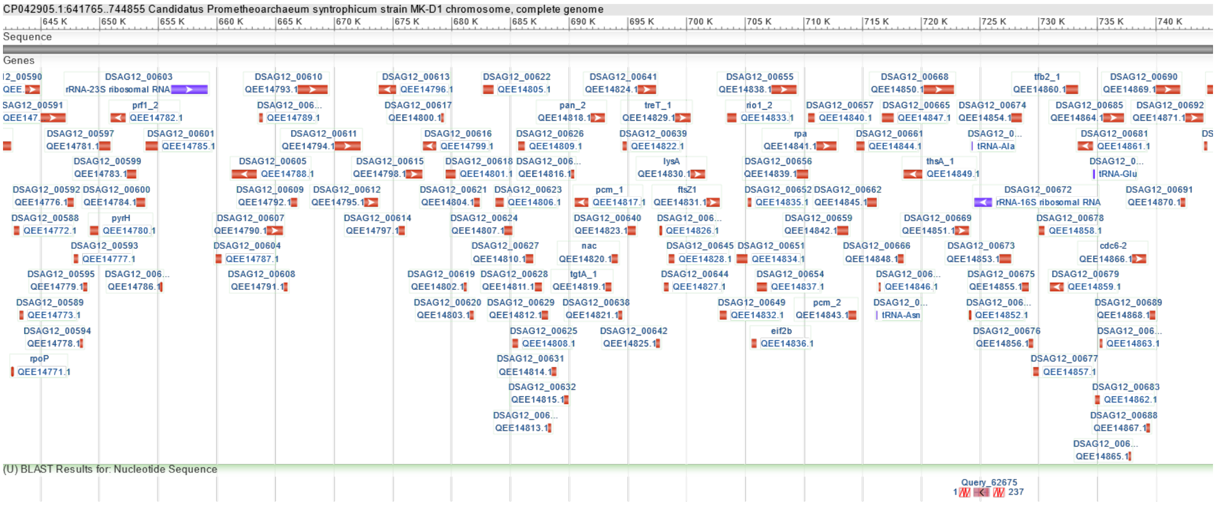

Candidatus Heimdallarchaeota archaeon AB_125

Overview:

https://img.jgi.doe.gov/cgi-bin/m/main.cgi?section=TaxonDetail&page=taxonDetail&taxon_oid=2740891990

16S rRNA

https://img.jgi.doe.gov/cgi-bin/m/main.cgi?section=TaxonDetail&page=rnas&taxon_oid=2740891990&locus_type=rRNA&gene_symbol=16S

- Scaffold Source:

https://img.jgi.doe.gov/cgi-bin/m/main.cgi?section=ScaffoldGraph&page=scaffoldGraph&scaffold_oid=2740901964&start_coord=1&end_coord=14619&marker_gene=2741395577&seq_length=14619

- Neighbourhood:

https://img.jgi.doe.gov/cgi-bin/m/main.cgi?section=GeneDetail&page=geneDetail&gene_oid=2741395578

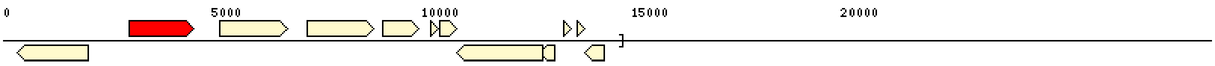

351-2030: Hypothetical protein

3022-4565: 16S rRNA

5183-6772: Hypothetical protein

7247-8836: Hypothetical protein

Heimdallarchaeota archaeon RS678

Overview:

https://img.jgi.doe.gov/cgi-bin/m/main.cgi?section=TaxonDetail&page=taxonDetail&taxon_oid=2778261486

16S rRNA

https://img.jgi.doe.gov/cgi-bin/m/main.cgi?section=TaxonDetail&page=rnas&taxon_oid=2778261486&locus_type=rRNA&gene_symbol=16S

- Scaffold source:

https://img.jgi.doe.gov/cgi-bin/m/main.cgi?section=ScaffoldGraph&page=scaffoldGraph&scaffold_oid=2778314286&start_coord=1&end_coord=19214&marker_gene=2780279842&seq_length=19214

- Neighbourhood:

https://img.jgi.doe.gov/cgi-bin/m/main.cgi?section=GeneDetail&page=geneDetail&gene_oid=2780279842

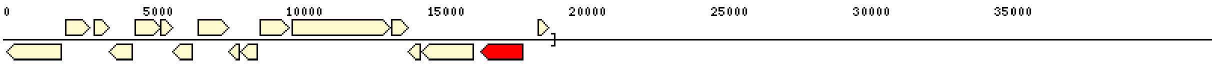

14792-16555: glutamyl-tRNA synthetase

16843-18311: 16S rRNA

18861-19214: hypothetical

23S rRNA

https://img.jgi.doe.gov/cgi-bin/m/main.cgi?section=TaxonDetail&page=rnas&taxon_oid=2778261486&locus_type=rRNA&gene_symbol=23S

- Scaffold Source:

https://img.jgi.doe.gov/cgi-bin/m/main.cgi?section=ScaffoldGraph&page=scaffoldGraph&scaffold_oid=2778314283&start_coord=1&end_coord=68485&marker_gene=2780279686&seq_length=68485

- Neighbourhood:

https://img.jgi.doe.gov/cgi-bin/m/main.cgi?section=GeneDetail&page=geneDetail&gene_oid=2780279686

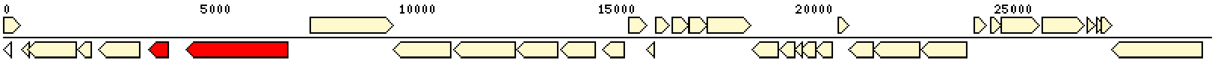

2417-3454: pyrophosphate-dependent phosphofructokinase

3663 -7182: 23S rRNA (3663-4177 + 4620-7182)

7737 – 9824: ssDNA-binding replication factor A large subunit

Candidatus Thorarchaeota archaeon AB_25

Overview:

https://img.jgi.doe.gov/cgi-bin/m/main.cgi?section=TaxonDetail&page=taxonDetail&taxon_oid=2775506787

16S rRNA

https://img.jgi.doe.gov/cgi-bin/m/main.cgi?section=TaxonDetail&page=rnas&taxon_oid=2775506787&locus_type=rRNA&gene_symbol=16S

- Scaffold source:

https://img.jgi.doe.gov/cgi-bin/m/main.cgi?section=ScaffoldGraph&page=scaffoldGraph&scaffold_oid=2775516521&start_coord=1&end_coord=3575&marker_gene=2775871763&seq_length=3575

- Neighbourhood:

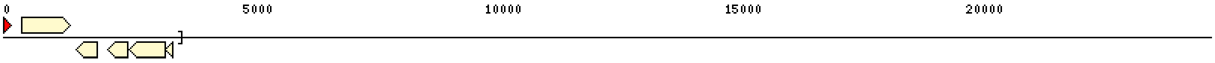

1-116 16S rRNA

395-1381: hypothetical

1532-1963: hypothetical

23S rRNA (no linked 2S9

https://img.jgi.doe.gov/cgi-bin/m/main.cgi?section=TaxonDetail&page=rnas&taxon_oid=2775506787&locus_type=rRNA&gene_symbol=23S

- Scaffold source:

https://img.jgi.doe.gov/cgi-bin/m/main.cgi?section=ScaffoldGraph&page=scaffoldGraph&scaffold_oid=2775516313&start_coord=1&end_coord=26908&marker_gene=2775869598&seq_length=26908

- Neighbourhood:

https://img.jgi.doe.gov/cgi-bin/m/main.cgi?section=GeneDetail&page=geneDetail&gene_oid=2775869598

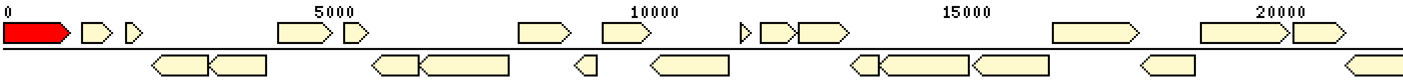

1-1060: 23S rRNA

1248-1727: hypothetical

1951-2208: hypothetical

5S rRNA

https://img.jgi.doe.gov/cgi-bin/m/main.cgi?section=TaxonDetail&page=rnas&taxon_oid=2775506787&locus_type=rRNA&gene_symbol=5S

- Scaffold source:

https://img.jgi.doe.gov/cgi-bin/m/main.cgi?section=ScaffoldGraph&page=scaffoldGraph&scaffold_oid=2775516322&start_coord=1&end_coord=22386&marker_gene=2775869808&seq_length=22386

- Neighbourhood:

https://img.jgi.doe.gov/cgi-bin/m/main.cgi?section=GeneDetail&page=geneDetail&gene_oid=2775869808

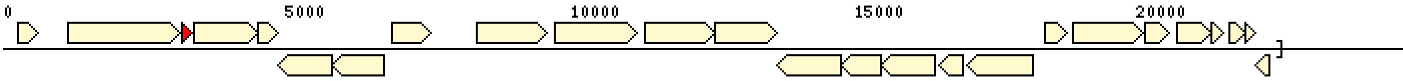

1152-3134 hypothetical

3161-3279: 5S rRNA

3355-4482: hypothetical

4490-4855: hypothetical

Candidatus Thorarchaeota archaeon SMTZ1-83

Overview:

https://img.jgi.doe.gov/cgi-bin/m/main.cgi?section=TaxonDetail&page=taxonDetail&taxon_oid=2740891981

16S rRNA

https://img.jgi.doe.gov/cgi-bin/m/main.cgi?section=TaxonDetail&page=rnas&taxon_oid=2740891981&locus_type=rRNA&gene_symbol=16S

16S rRNA #1 (no linked 23/5S)

- Scaffold source:

https://img.jgi.doe.gov/cgi-bin/m/main.cgi?section=ScaffoldGraph&page=scaffoldGraph&scaffold_oid=2740900874&start_coord=1&end_coord=5948&marker_gene=2741370659&seq_length=5948

- Neighbourhood:

https://img.jgi.doe.gov/cgi-bin/m/main.cgi?section=GeneDetail&page=geneDetail&gene_oid=2741370659

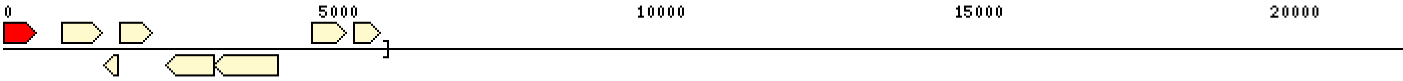

1-516: 16S rRNA

945-1565: protein-S-isoprenylcysteine O-methyltransferase

1589-1801 hypothetical

16S rRNA #2 (inconclusive)

- Scaffold source:

https://img.jgi.doe.gov/cgi-bin/m/main.cgi?section=ScaffoldGraph&page=scaffoldGraph&scaffold_oid=2740901004&start_coord=1&end_coord=12722&marker_gene=2741372168&seq_length=12722

- Neighbourhood:

https://img.jgi.doe.gov/cgi-bin/m/main.cgi?section=GeneDetail&page=geneDetail&gene_oid=2741372168

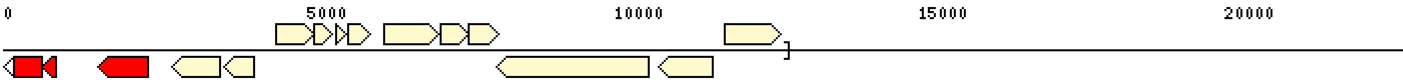

23S rRNA (no linked 23S/5S)

https://img.jgi.doe.gov/cgi-bin/m/main.cgi?section=TaxonDetail&page=rnas&taxon_oid=2740891981&locus_type=rRNA&gene_symbol=23S

- Scaffold source:

https://img.jgi.doe.gov/cgi-bin/m/main.cgi?section=ScaffoldGraph&page=scaffoldGraph&scaffold_oid=2740900796&start_coord=1&end_coord=24031&marker_gene=2741369673&seq_length=24031

- Neighbourhood:

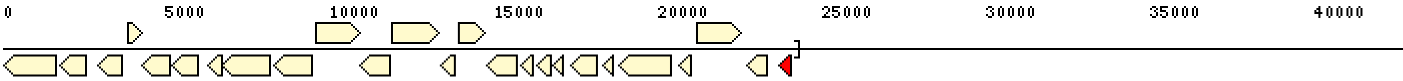

22651-23277: hypothetical

23639-24031: 23S rRNA

Wukongarchaeote WOR431 - LMSG_G000000612.1

Source: https://www.biosino.org/elmsg/record/MSG071613

Query sequence 16S and 23S rRNA from *Ca.* Prometheoarchaeum syntrophicum

Wukong 16S rRNA on contig_4272291: 3´end of 16S rRNA not included (end of contig) – not conclusive

Wukong 23S RNA on contig_681832: no 16S rRNA detected within the ~ 7kb upstream of 23S rRNA (Contig size 24392 bp)

Wukongarchaeote WOR133 - LMSG_G000000613.1

Source: https://www.biosino.org/elmsg/record/MSG071614

Query sequence: 16S and 23S rRNA from *Ca.* Prometheoarchaeum syntrophicum and Wukongarchaeote WOR431

Wukong 16S rRNA on contig: unknown

Wukong 23S RNA on contig_2017418 (18783 bp) no 16S rRNA detected within the >7 kb upstream of 23S rRNA

Njordarchaeote GB154

Source: https://www.biosino.org/elmsg/record/MSG071612

Query sequence: 16S and 23S rRNA from *Ca.* Prometheoarchaeum syntrophicum and Wukongarchaeote WOR431

16S rRNA on contig_2337563

23S rRNA on contig_4560732 (size 51154 bp) (23S located between ~ 24-27 kb), no matching with 16S in contig_2337563, 16S rRNA from *Ca.* Prometheoarchaeum syntrophicum and Wukongarchaeote WOR43

Njordarchaeote GB136

Source: https://www.biosino.org/elmsg/record/MSG071611

16S rRNA unclear

23S rRNA on contig_199659 (size 5396 bp) (only partial sequence found 3´en 23S at the 5 ´end of the contig).

Sigynarchaeote SQRJ88

Source: https://www.biosino.org/elmsg/record/MSG071617

23S rRNA contig_1571010 (size 58759 bp) … no 16S rRNA detected in 3kb upstream

16S rRNA (partial) contig_558645 (size 29383): unclear 16S but no 23S in contig

Candidatus Odinarchaeota archaeon LCB_4

Overview:

https://img.jgi.doe.gov/cgi-bin/m/main.cgi?section=TaxonDetail&page=taxonDetail&taxon_oid=2740891986

16S rRNA

https://img.jgi.doe.gov/cgi-bin/m/main.cgi?section=GeneDetail&page=geneDetail&gene_oid=2741388754

23S rRNA

https://img.jgi.doe.gov/cgi-bin/m/main.cgi?section=GeneDetail&page=geneDetail&gene_oid=2741388755

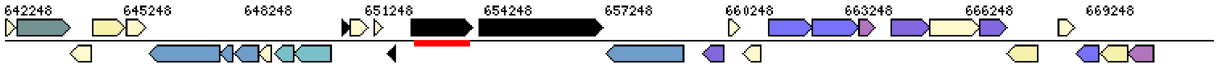

16S and 23S rRNA are indicated in black

Nanoarchaeum equitans Kin4-M chromosome, complete genome

Source GenBank: AE017199

16S rRNA

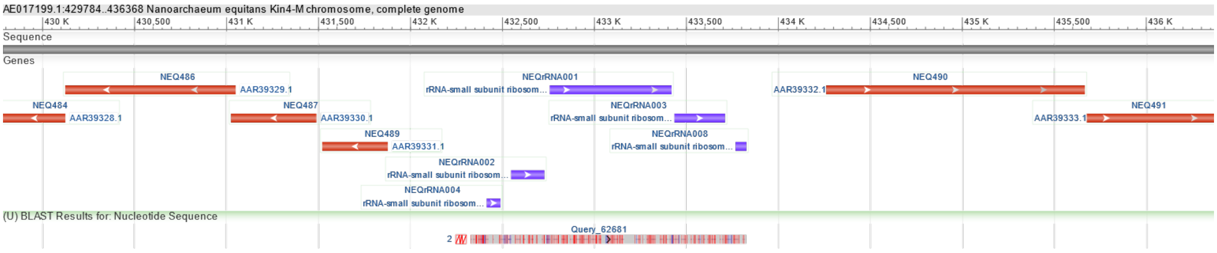

23S rRNA

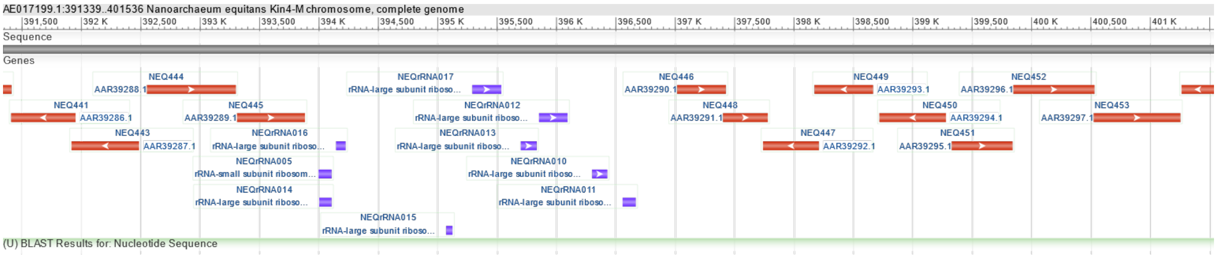

Candidatus Micrarchaeum sp. strain A_DKE chromosome, complete genome

NCBI Reference Sequence: NZ_CP060530.1

16S rRNA

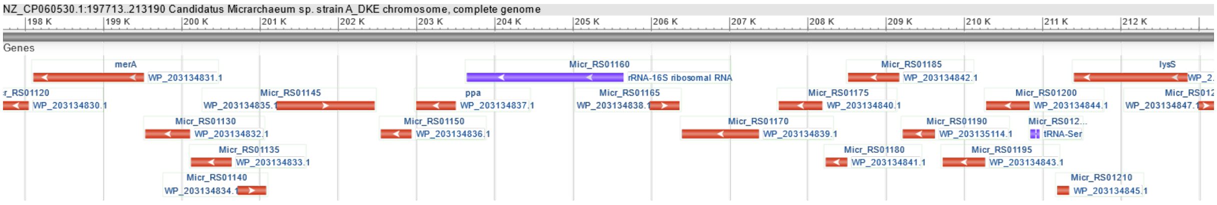

23S rRNA

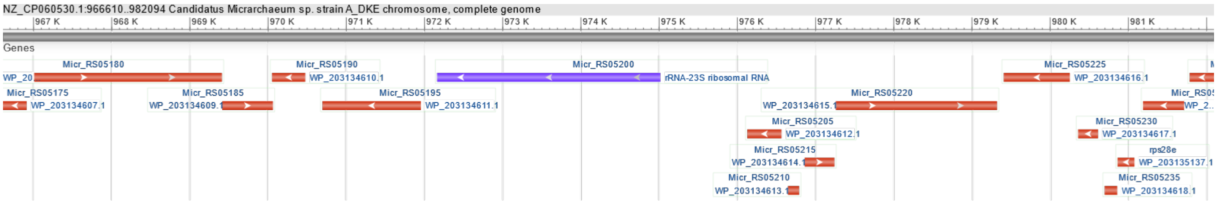

Candidatus Nanohalobium constans strain LC1Nh chromosome, complete genome

NCBI Reference Sequence: NZ_CP040089.1

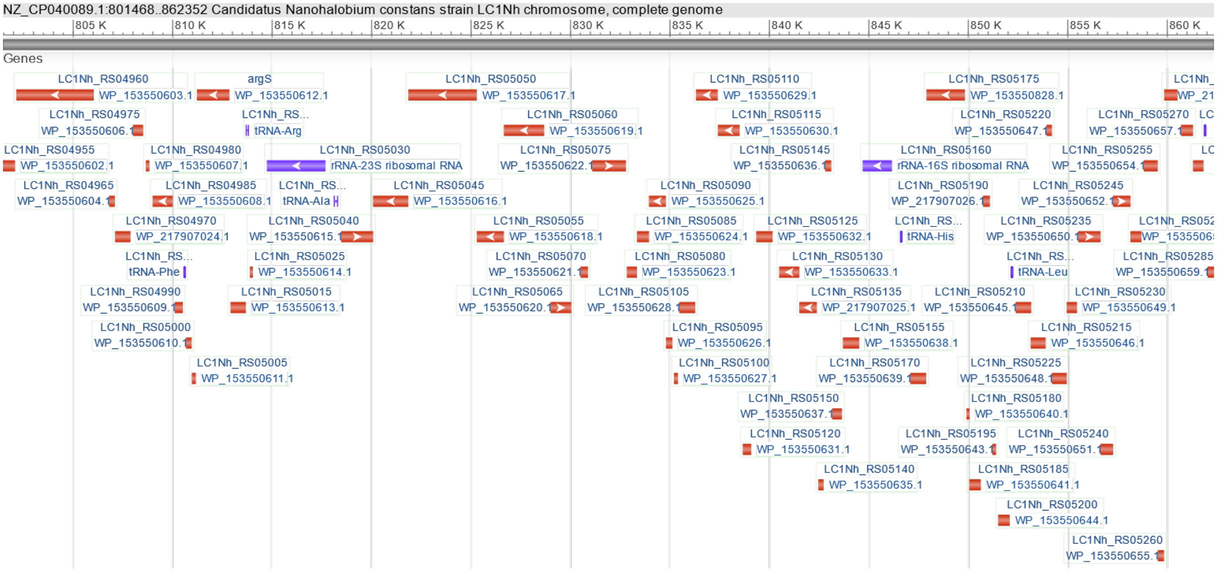

Ferroplasma acidarmanus fer1, complete genome

GenBank: CP004145.1

16S rRNA

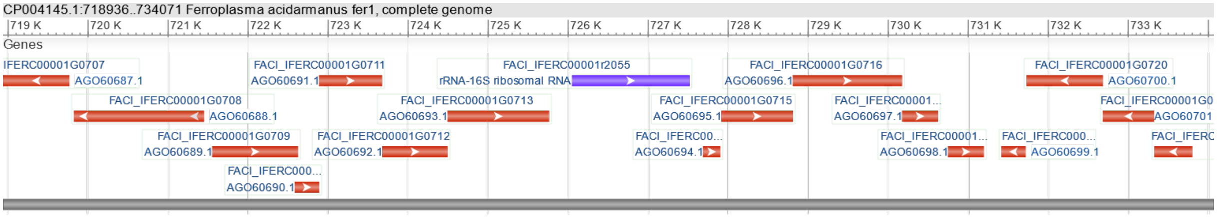

23S rRNA

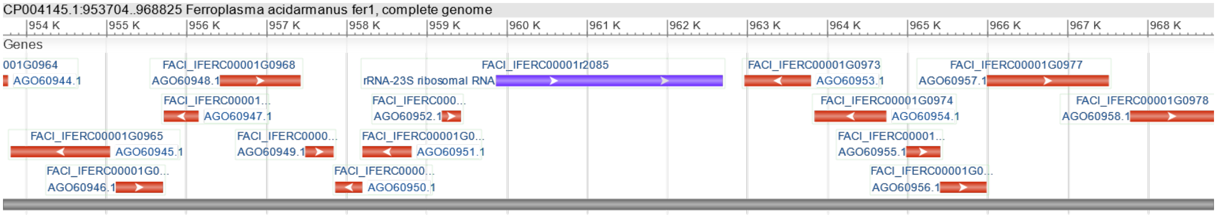

Ignicoccus hospitalis KIN4/I, complete genome

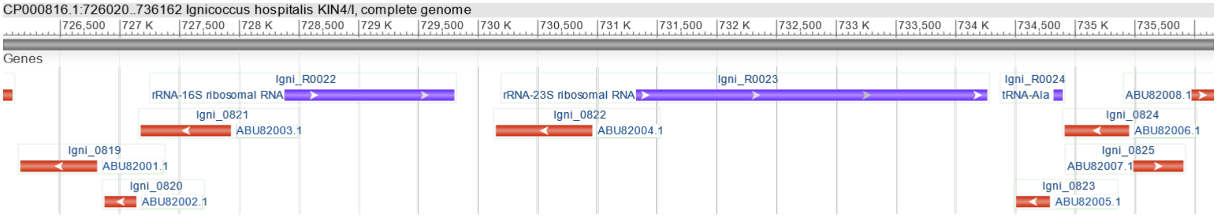

Candidatus Woesearchaeota archaeon isolate NC_groundwater_85_Ag_S-0.1um_31_13 chromosome

GenBank: CP066683.1

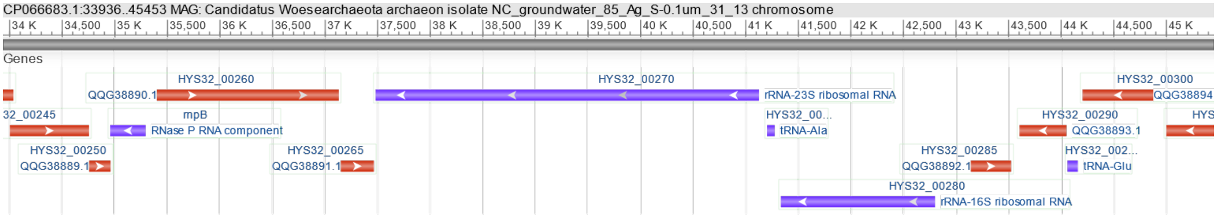

Woesearchaeota archaeon ARS106

https://img.jgi.doe.gov/cgi-bin/m/main.cgi?section=TaxonDetail&page=taxonDetail&taxon_oid=2785511140

16S+23S

https://img.jgi.doe.gov/cgi-bin/m/main.cgi?section=ScaffoldGraph&page=scaffoldGraph&scaffold_oid=2785544388&start_coord=1&end_coord=62559&marker_gene=2786194161&seq_length=62559

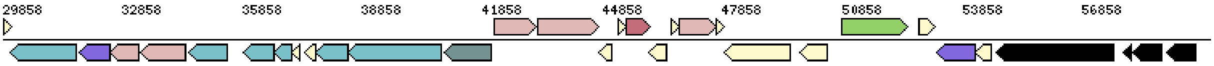

23S- tRNA-16S rRNA in black

Candidatus Aenigmarchaeota archaeon isolate NC_groundwater_134_Ag_S-0.1um_56_17 chromosome

GenBank: CP066684.1

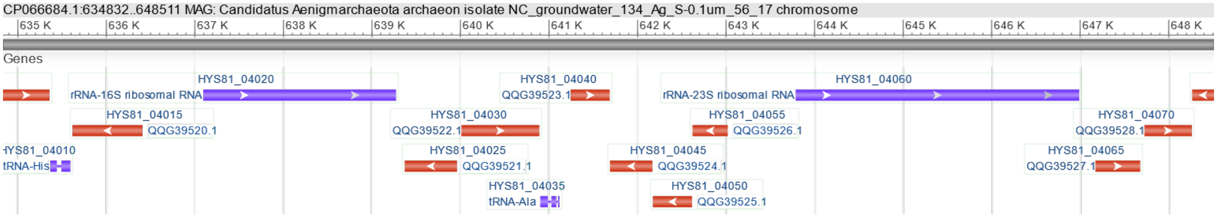

Candidatus Aenigmarchaeota archaeon LHA6

https://img.jgi.doe.gov/cgi-bin/m/main.cgi?section=TaxonDetail&page=taxonDetail&taxon_oid=2875759356

16+23S rRNA

https://img.jgi.doe.gov/cgi-bin/m/main.cgi?section=ScaffoldGraph&page=scaffoldGraph&scaffold_oid=2875759356&start_coord=1&end_coord=27007&marker_gene=2875759361&seq_length=27007

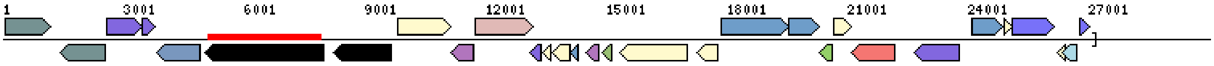

in black 23S-16S (no tRNA Ala?)

Candidatus Aenigmarchaeota archaeon LHD6

https://img.jgi.doe.gov/cgi-bin/m/main.cgi?section=TaxonDetail&page=taxonDetail&taxon_oid=2875931624

16+23S rRNA

https://img.jgi.doe.gov/cgi-bin/m/main.cgi?section=ScaffoldGraph&page=scaffoldGraph&scaffold_oid=2875931638&start_coord=1&end_coord=9655&marker_gene=2875932010&seq_length=9655

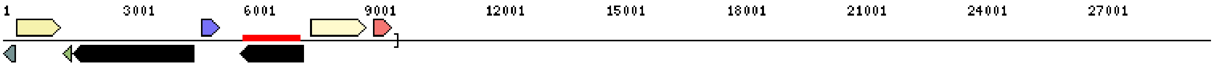

in black 23S/16S rRNA (no tRNA Ala?)

1744-4748: 23S rRNA

4934-5380: membrane-associated phospholipid phosphatase

5905-7456: 16S rRNA

Candidatus Diapherotrites archaeon isolate Fred_18-Q3-R57-64_BAT3C.431 chromosome, complete genome

GenBank: CP064981.1

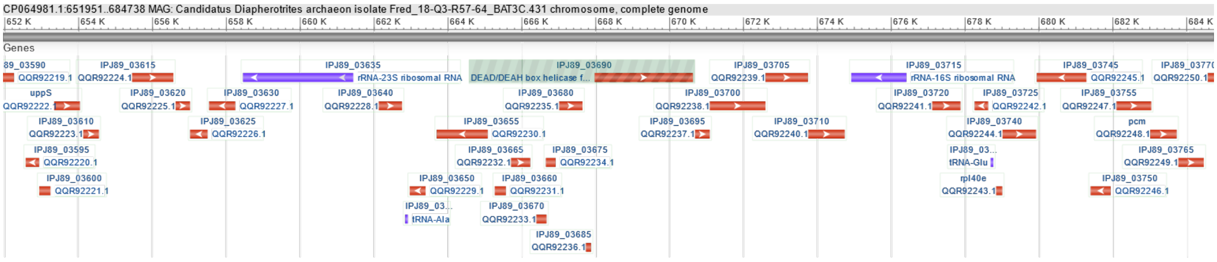

Candidatus Nanosalinarum sp. J07AB56

16S rRNA

https://img.jgi.doe.gov/cgi-bin/m/main.cgi?section=TaxonDetail&page=rnas&taxon_oid=2502422327&locus_type=rRNA&gene_symbol=16S

- Scaffold source:

https://img.jgi.doe.gov/cgi-bin/m/main.cgi?section=ScaffoldGraph&page=scaffoldGraph&scaffold_oid=2502426305&start_coord=381094&end_coord=782580&marker_gene=2502522245&seq_length=959093

- Neighbourhood:

https://img.jgi.doe.gov/cgi-bin/m/main.cgi?section=GeneDetail&page=geneDetail&gene_oid=2502522245

-

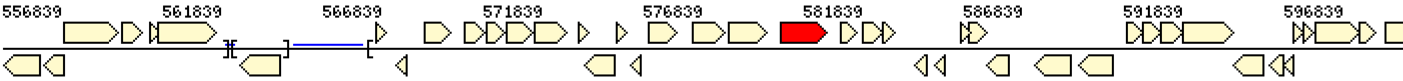

579466-580686: Glutamate dehydrogenase/leucine dehydrogenase

581094-582580: 16S rRNA

582980-583507: hypothetical

23S rRNA

https://img.jgi.doe.gov/cgi-bin/m/main.cgi?section=TaxonDetail&page=rnas&taxon_oid=2502422327&locus_type=rRNA&gene_symbol=23S

- Scaffold source:

https://img.jgi.doe.gov/cgi-bin/m/main.cgi?section=ScaffoldGraph&page=scaffoldGraph&scaffold_oid=2502426304&start_coord=1&end_coord=60285&marker_gene=2502521495&seq_length=60285

- Neighbourhood:

https://img.jgi.doe.gov/cgi-bin/m/main.cgi?section=GeneDetail&page=geneDetail&gene_oid=2502521495

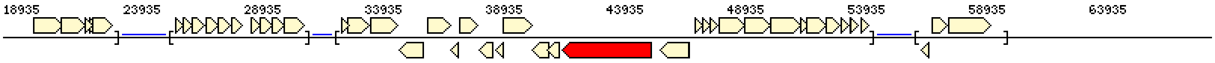

41497-41961: cytidine deaminase

42089-45778: 23S rRNA

46139-47326: Cytochrome c biogenesis protein

Candidatus Altiarchaeales archaeon WOR_SM1_86-2

Overview:

https://img.jgi.doe.gov/cgi-bin/m/main.cgi?section=TaxonDetail&page=taxonDetail&taxon_oid=2740891980

16S rRNA

https://img.jgi.doe.gov/cgi-bin/m/main.cgi?section=TaxonDetail&page=rnas&taxon_oid=2740891980&locus_type=rRNA&gene_symbol=16S

- Scaffold source:

https://img.jgi.doe.gov/cgi-bin/m/main.cgi?section=ScaffoldGraph&page=scaffoldGraph&scaffold_oid=2740900703&start_coord=1&end_coord=30582&marker_gene=2741368404&seq_length=30582

- Neighbourhood:

https://img.jgi.doe.gov/cgi-bin/m/main.cgi?section=GeneDetail&page=geneDetail&gene_oid=2741368404

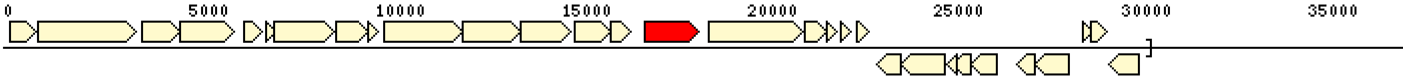

16274-16828: arginine decarboxylase

14193-18661: 16S rRNA

18927-21428: hypothetical

23S rRNA

https://img.jgi.doe.gov/cgi-bin/m/main.cgi?section=TaxonDetail&page=rnas&taxon_oid=2740891980&locus_type=rRNA&gene_symbol=23S

- Scaffold source:

https://img.jgi.doe.gov/cgi-bin/m/main.cgi?section=ScaffoldGraph&page=scaffoldGraph&scaffold_oid=2740900624&start_coord=1&end_coord=20366&marker_gene=2741367329&seq_length=20366

- Neighbourhood:

https://img.jgi.doe.gov/cgi-bin/m/main.cgi?section=GeneDetail&page=geneDetail&gene_oid=2741367329

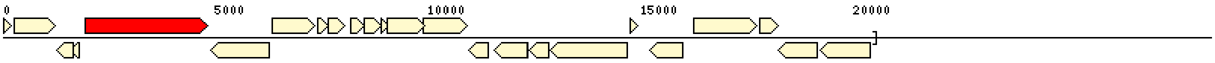

1243-1784: thymidylate kinase

1928-4816: 23S rRNA

4869-6269: RecJ-like exonuclease

Candidate division Huberarchaea archaeon CG_4_9_14_0_8_um_filter_31_21

https://img.jgi.doe.gov/cgi-bin/m/main.cgi?section=TaxonDetail&page=taxonDetail&taxon_oid=2785510816

16S rRNA

https://img.jgi.doe.gov/cgi-bin/m/main.cgi?section=TaxonDetail&page=rnas&taxon_oid=2785510816&locus_type=rRNA&gene_symbol=16S

- Scaffold source:

https://img.jgi.doe.gov/cgi-bin/m/main.cgi?section=ScaffoldGraph&page=scaffoldGraph&scaffold_oid=2785515709&start_coord=1&end_coord=12278&marker_gene=2785880803&seq_length=12278

- Neighbourhood:

https://img.jgi.doe.gov/cgi-bin/m/main.cgi?section=GeneDetail&page=geneDetail&gene_oid=2785880803

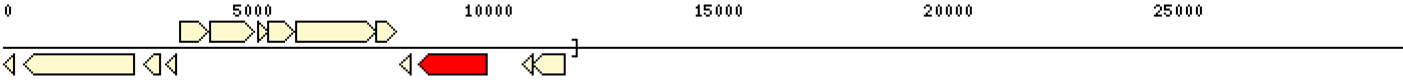

Candidatus Pacearchaeota archaeon CG10 (Unlinked? – ambiguous) Unusual long spacers

https://img.jgi.doe.gov/cgi-bin/m/main.cgi?section=TaxonDetail&page=taxonDetail&taxon_oid=2785510784

- Scaffold source:

https://img.jgi.doe.gov/cgi-bin/m/main.cgi?section=ScaffoldGraph&page=scaffoldGraph&scaffold_oid=2785514001&start_coord=1&end_coord=189970&marker_gene=2785853019&seq_length=189970

- Neighbourhood:

23S rRNA https://img.jgi.doe.gov/cgi-bin/m/main.cgi?section=GeneDetail&page=geneDetail&gene_oid=2785853019

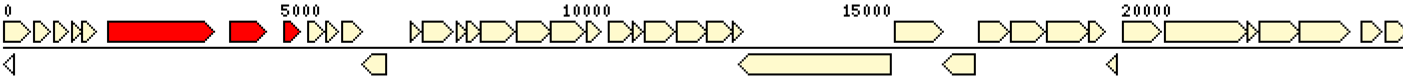

16S rRNA https://img.jgi.doe.gov/cgi-bin/m/main.cgi?section=GeneDetail&page=geneDetail&gene_oid=2785853014

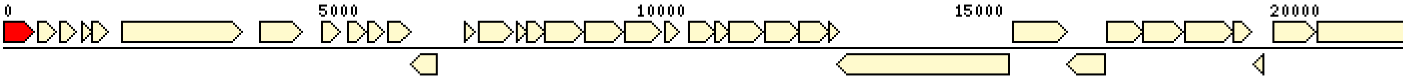

Annotation error/Unusual long spacers with small hypothetical ORF (see Pacearchaeota archaeon SCGC AG-274-K21)

1-494: 16S rRNA
560-850: hypothetical
895-1164: hypothetical
1242-1312: tRNA Ala

1405-1656: hypothetical
1876-5304: 23S rRNA
5545-5720: hypothetical

Pacearchaeota archaeon SCGC AG-274-K21

https://img.jgi.doe.gov/cgi-bin/m/main.cgi?section=TaxonDetail&page=taxonDetail&taxon_oid=2716884242

16S and 23S rRNA

https://img.jgi.doe.gov/cgi-bin/m/main.cgi?section=TaxonDetail&page=rnas&taxon_oid=2716884242&locus_type=rRNA

https://img.jgi.doe.gov/cgi-bin/m/main.cgi?section=ScaffoldGraph&page=scaffoldGraph&scaffold_oid=2716894039&start_coord=1&end_coord=29909&marker_gene=2717095016&seq_length=29909

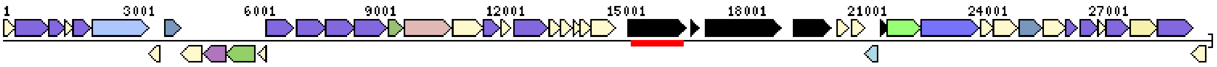

in black 16S – tRNA Ala – 23S rRNA

Similar organisation in Pacearchaeota archaeon NP1466 (probably including annotation error overlapping with tRNA Ala).

https://img.jgi.doe.gov/cgi-bin/m/main.cgi?section=TaxonDetail&page=taxonDetail&taxon_oid=2811995088

Aigarchaeota archaeon SCGC AAA471-J07

https://img.jgi.doe.gov/cgi-bin/m/main.cgi?section=TaxonDetail&page=taxonDetail&taxon_oid=2264867226

16S rRNA

- Scaffold source:

https://img.jgi.doe.gov/cgi-bin/m/main.cgi?section=ScaffoldGraph&page=scaffoldGraph&scaffold_oid=2264886741&start_coord=1&end_coord=33878&marker_gene=2265091874&seq_length=33878

- Neighbourhood:

https://img.jgi.doe.gov/cgi-bin/m/main.cgi?section=GeneDetail&page=geneDetail&gene_oid=2265091874

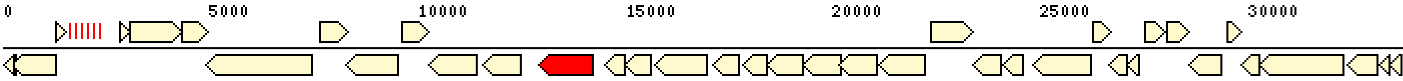

11546-15445: hypothetical

12899-14221: 16S rRNA

14468-14947: NADH-quinone oxidoreductase subunit C

Candidatus Caldiarchaeum subterraneum DNA, complete genome,

GenBank: BA000048.1

16S rRNA

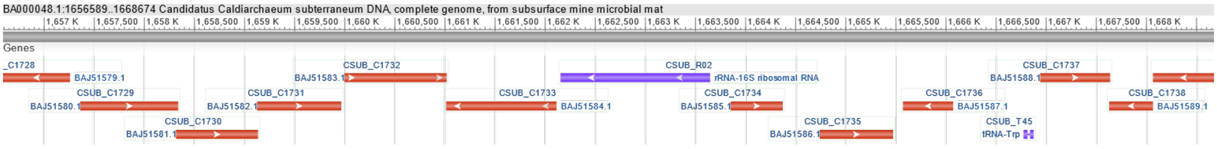

23S rRNA

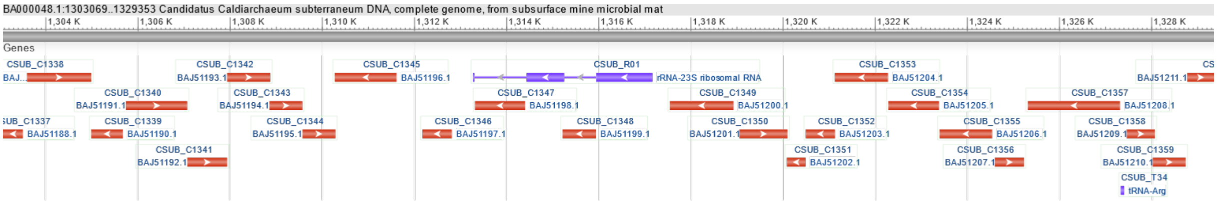

Candidatus Nitrososphaera gargensis Ga9-2

https://img.jgi.doe.gov/cgi-bin/m/main.cgi?section=TaxonDetail&page=taxonDetail&taxon_oid=2510065023

16S+23S rRNA

https://img.jgi.doe.gov/cgi-bin/m/main.cgi?section=ScaffoldGraph&page=scaffoldGraph&scaffold_oid=2510066862&start_coord=757278&end_coord=1210240&marker_gene=2510153226&seq_length=2833736

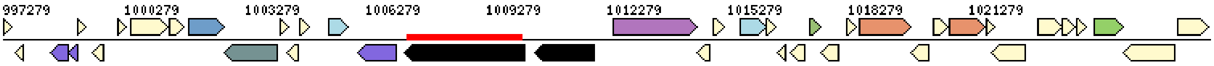

in black 23S-16S rRNA

***Nitrososphaera viennensis* EN76, complete genome (unlinked? very small ORF annotated in between -annotation issue?!)**

GenBank: CP007536.1

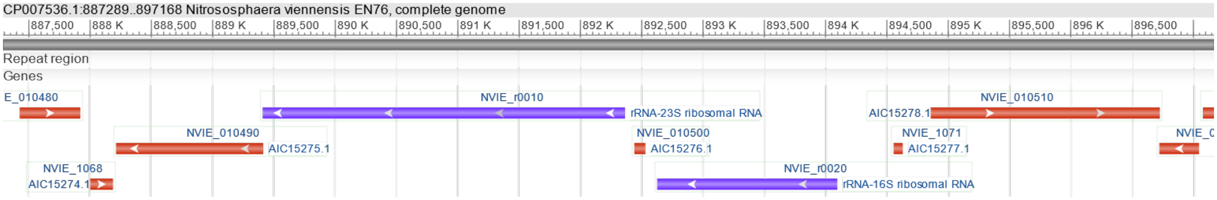

Nitrosopumilus sp. Nsub

https://img.jgi.doe.gov/cgi-bin/m/main.cgi?section=TaxonDetail&page=taxonDetail&taxon_oid=2690315682

- Scaffold source:

https://img.jgi.doe.gov/cgi-bin/m/main.cgi?section=ScaffoldGraph&page=scaffoldGraph&scaffold_oid=2690318230&start_coord=1&end_coord=52337&marker_gene=2690705878&seq_length=52337

16S +23S

https://img.jgi.doe.gov/cgi-bin/m/main.cgi?section=ScaffoldGraph&page=scaffoldGraph&scaffold_oid=2690318230&start_coord=1&end_coord=52337&marker_gene=2690705878&seq_length=52337

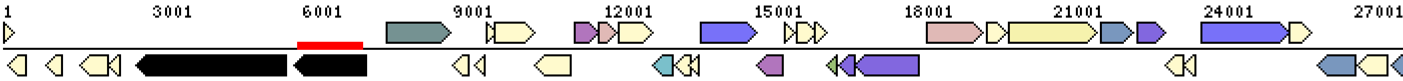

23S and 16S rRNA in black

Haloferax volcanii DS2, complete sequence

GenBank: CP001956.1

rDNA#1

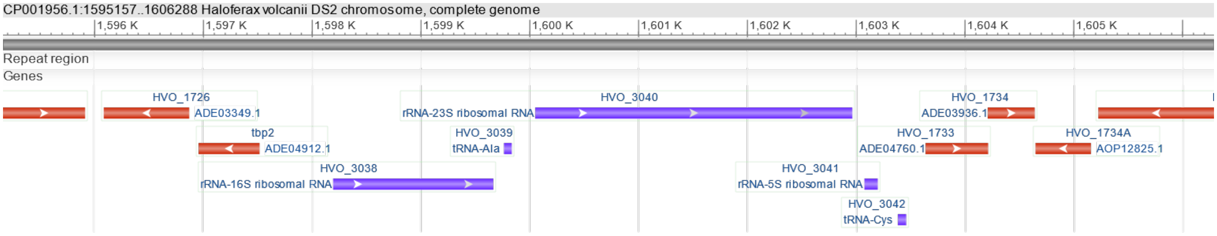

rDNA#2

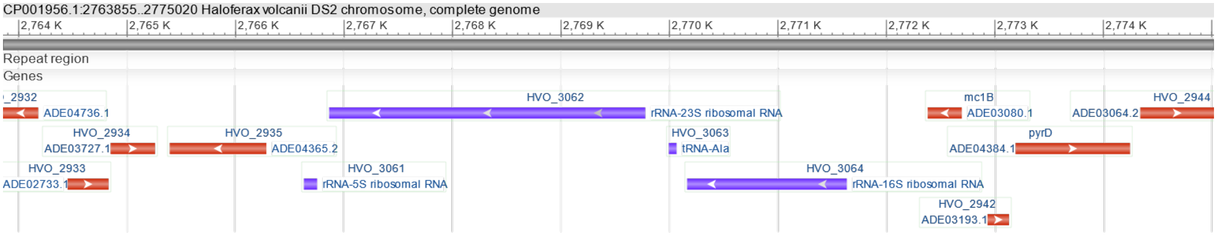

Sulfolobus acidocaldarius strain NG05B_CO5_07 genome

GenBank: CP013695.1

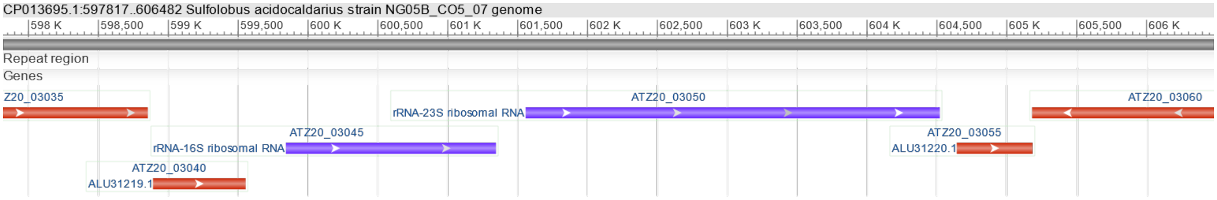

Candidatus Korarchaeum cryptofilum OPF8, complete genome

GenBank: CP000968.1

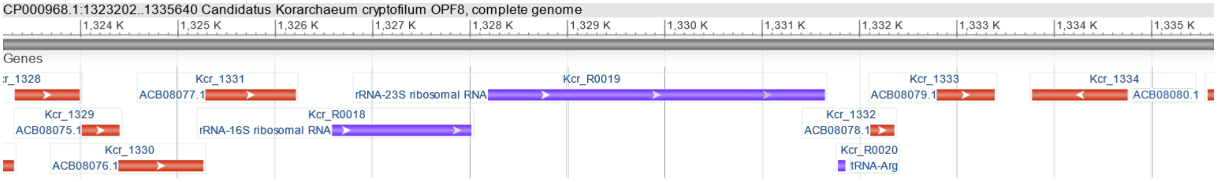

Methanonatronarchaeum thermophilum AMET1

https://img.jgi.doe.gov/cgi-bin/m/main.cgi?section=TaxonDetail&page=taxonDetail&taxon_oid=2786546529

16+23S rRNA

https://img.jgi.doe.gov/cgi-bin/m/main.cgi?section=ScaffoldGraph&page=scaffoldGraph&scaffold_oid=2786603347&start_coord=1&end_coord=224098&marker_gene=2787456131&seq_length=483384

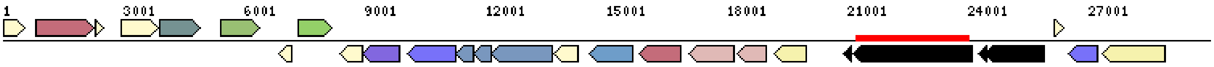

in black 23S-tRNA Ala-16S

Candidatus Methanoperedens nitroreducens ANME-2d (ANME_V10)

https://img.jgi.doe.gov/cgi-bin/m/main.cgi?section=TaxonDetail&page=taxonDetail&taxon_oid=2515154041

16S+23S rRNA

https://img.jgi.doe.gov/cgi-bin/m/main.cgi?section=ScaffoldGraph&page=scaffoldGraph&scaffold_oid=2515157734&start_coord=261866&end_coord=544976&marker_gene=2515322110&seq_length=544976

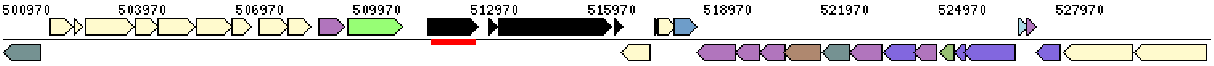

in black 16S-tRNA Ala-23S-5S rRNA

***Thermoproteus tenax* Kra 1, complete genome**

GenBank: FN869859.1

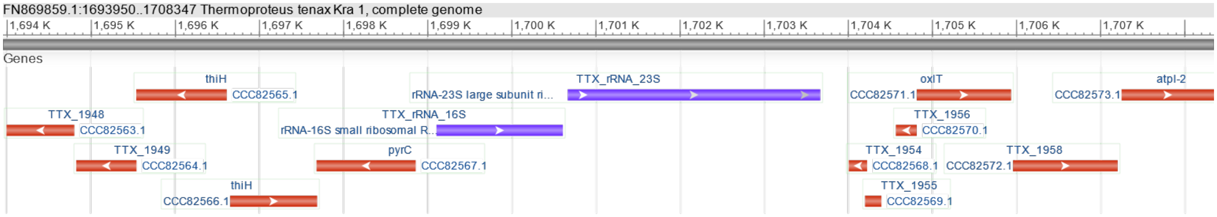

Supplement: msac054_Supplementary_Data [file msac054_supplementary_data.pdf]
